# Spinach (*Spinacia oleracea* L.) Growth Model in Indoor Controlled Environment Using Agriculture 4.0

**DOI:** 10.3390/s25061684

**Published:** 2025-03-08

**Authors:** Cesar Isaza, Angel Mario Aleman-Trejo, Cristian Felipe Ramirez-Gutierrez, Jonny Paul Zavala de Paz, Jose Amilcar Rizzo-Sierra, Karina Anaya

**Affiliations:** Cuerpo Académico de Tecnologías de la Información y Comunicación Aplicada, Universidad Politécnica de Querétaro, Carretera Estatal 420 SN, El Marqués 76240, Querétaro, Mexico; cesar.isaza@upq.edu.mx (C.I.); ein_angel@hotmail.com (A.M.A.-T.); cristian.ramirez@upq.edu.mx (C.F.R.-G.); jonny.zavala@upq.edu.mx (J.P.Z.d.P.); jose.rizzo@upq.edu.mx (J.A.R.-S.)

**Keywords:** Viroflay spinach (*Spinacia oleracea* L.), plant growth model, Agriculture 4.0, regression models

## Abstract

Global trends in health, climate, and population growth drive the demand for high-nutrient plants like spinach, which thrive under controlled conditions with minimal resources. Despite technological advances in agriculture, current systems often rely on traditional methods and need robust computational models for precise plant growth forecasting. Optimizing vegetable growth using advanced agricultural and computational techniques, addressing challenges in food security, and obtaining efficient resource utilization within urban agriculture systems are open problems for humanity. Considering the above, this paper presents an enclosed agriculture system for growth and modeling spinach of the Viroflay (*Spinacia oleracea* L.) species. It encompasses a methodology combining data science, machine learning, and mathematical modeling. The growth system was built using LED lighting, automated irrigation, temperature control with fans, and sensors to monitor environmental variables. Data were collected over 60 days, recording temperature, humidity, substrate moisture, and light spectra information. The experimental results demonstrate the effectiveness of polynomial regression models in predicting spinach growth patterns. The best-fitting polynomial models for leaf length achieved a minimum Mean Squared Error (MSE) of 0.158, while the highest MSE observed was 1.2153, highlighting variability across different leaf pairs. Leaf width models exhibited improved predictability, with MSE values ranging from 0.0741 to 0.822. Similarly, leaf stem length models showed high accuracy, with the lowest MSE recorded at 0.0312 and the highest at 0.3907.

## 1. Introduction

The importance of vegetables in a daily diet has become an issue of fundamental importance not only to improve the health of the global population but also to deal with the growing problem of famine. Recently, global warming and political, economic, and social tendencies have forced all governments to plan and project strategies to ensure food for all citizens in the coming decades. Consequently, new technologies are required to deal with the alarming demands for food. Several tendencies have appeared, such as agriculture systems using less water, increasing production based on greenhouse-growing processes, or selecting more energetical vegetables that use fewer resources.

Agriculture systems and the food industry must improve the production of vegetables like lettuce, cabbage, eggplant, green beans, tomatoes, or spinach [1]. The last one has excellent properties like a good amount of calories, low-fat content, carbs, and high protein [2,3]. In addition, spinach (*Spinacia oleracea* L.) can be grown with minimal resources compared to other vegetables. In 2015, Zhang et al. [4] reported that optimal water levels for spinach growth ranged between 36.15 cm and 42 cm, representing lower values compared to traditional field irrigation, which often results in excessive water use and nutrient leaching. The authors highlight that spinach can be cultivated with significantly lower water input while achieving comparable or superior biomass production, reinforcing the potential of precision agriculture to enhance resource efficiency in vegetable production.

Considering the above, the development of new technology to develop, monitor, and predict the plant development of this kind of vegetable is worthy of investigation.

Technically, the improvement of agriculture systems uses known paradigms: large amounts of land, unique soil, an irrigation system that demands a lot of water, controlled environmental variables, expensive greenhouse infrastructure, and the expertise of a grower who is in charge of making all decisions based on the accumulated experience in many crop cycles of the same plant species [5,6]. Even if promoting this kind of spinach-growing system is possible, it is mainly feasible in rural areas. Thus, another issue appears regarding the logistics required by transportation, distribution, and retail structures [7].

On the other hand, modern human lifestyle has made a general movement from rural to urban areas in the last century. Thus, the rising population of cities is reducing the land available to produce food. Therefore, recent advances in agriculture have appeared to grow food in small areas like vertical farming and roof garden systems [8,9,10]. However, these systems still use the same method of agriculture based on growers’ expertise, and only simple electronic devices have been proposed to assist production. Generally, humidity, substrate, ambient temperatures, and lighting control conditions have been used [11].

Considering the above, this research attempts to deal with the problem of modeling the natural development process of the spinach plant growing in indoor controlled environments and using Agriculture 4.0 tools [12]. We propose using a simple dry vertical infrastructure with optimal plant volume distribution, a low-cost electronic system to monitor and collect data, and a detailed model to track the growth of the plant.

Thus, a detailed spinach growth model was developed using regression models, considering the complex development of individual leaf pairs, stem length, and other key attributes. Building upon this foundation, the present study makes several key contributions:

1. Construct an enclosed, automated indoor cultivation system designed to foster the growth of spinach plants while ensuring freedom from spore, bacterial, and viral infections and simultaneously optimizing the utilization of agricultural resources, namely, water and energy.

2. Apply advanced machine learning algorithms to create a robust database for high-precision monitoring of spinach growth.

3. Develop an artificial intelligence model that learns from spinach growth patterns, enhancing automation and improving diagnostic capabilities within the enclosed cultivation system. This AI-driven approach aims to maximize food production efficiency in urban agricultural settings.

To comprehensively address these objectives, this paper is structured as follows: Section 2 reviews the most relevant literature on Agriculture 4.0, highlighting advancements in precision farming and controlled-environment agriculture. Section 3 outlines the materials and methods, detailing the sensor-equipped agricultural systems, IoT-based data collection techniques, and image-processing approaches used in the study. Section 4 presents the experimental results, evaluating different growth models and their predictive accuracy. Finally, the paper concludes with a discussion of the implications of the research for future agricultural technologies and food security solutions.

## 2. Related Works

### 2.1. Agriculture 4.0 and IoT

Despite several technologies developed for optimizing agriculture systems, most are based on electronic devices to monitor and act automatically in an on/off way [13]. Other researchers have proposed using specific environmental and nutrition variables to grow spinach in greenhouses or outdoors [14]. However, a mathematical model implemented in the form of a computation tool for following and forecasting the state of the leaf in spinach plants is still missing.

Computational plant models are significant because they describe the complex relationships between different seeds obtained with genetic modification, growing conditions, and plant development. Despite the advantages of this knowledge, it is not always easy to systematically obtain information using software or electronic devices due to the complexity of agriculture systems and the plant itself. However, any contribution to this issue will directly impact the understanding and improvement of agriculture technology to contribute to the problem of food assurance.

Artificial intelligence applied to agriculture can be found as part of the Industrial Revolution 4.0. There are commercially available robots for farming and automatic sensing with uncrewed aerial vehicles (UAVs). Recently, it has been possible to monitor, diagnose, and forecast the state of an entire crop. However, electronics for vertical farming and roof growing systems with machine learning and well-tested mathematical models are too constrained. Thus, the current study deals with the complex problem of modeling the natural development of spinach plants with high accuracy to build better agriculture systems based on current technologies like artificial intelligence.

In 2012, Takakavok et al. examined the impact of LED lighting on plant photomorphogenesis, breeding cycles, and growth optimization in Brassica juncea, Lactuca sativa, Ocimum gratissimum, Coleus blumei, and Tagetes patula. The experiments were conducted in controlled environments, with plants initially grown in greenhouses before being exposed to LEDs emitting at 460 nm (blue), 635 nm (short-wave red), and 660 nm (long-wave red) under an 18 h photoperiod with a PPF of 170 ± 10 µmol m^−2^s^−1^. Results showed that light spectral quality significantly affected plant development—Indian mustard experienced delayed flowering under 660 nm, while Coleus exhibited increased biomass with 460 nm and 635 nm LEDs. Growth parameters like leaf number, shoot weight, and leaf area varied significantly, with some reductions under excessive long-wave red light, likely due to shade-avoidance responses [7].

Similarly, Piovene et al. examined the impact of red-to-blue LED light ratios on growth, productivity, and nutritional quality in sweet basil and strawberries under controlled indoor conditions. The authors used five light treatments (red: blue ratios from 0.7 to 5.5), and a fluorescent control was tested. Basil biomass was highest at 0.7 but decreased by 16–68% at higher ratios, while strawberry yield tripled under optimized LED conditions (0.7 and 1.1 ratios). Experiments showed that the energy use efficiency improved up to nine-fold, and LED lighting enhanced the antioxidant content in basil while reducing nitrate accumulation [15].

In 2016, Ferrandez-Pastor et al. studied the application of information technologies in precision agriculture [16]. The authors developed a low-cost, energy-efficient IoT-based sensor network for precision agriculture, focusing on a greenhouse hydroponic system. Data acquisition was achieved with sensors to monitor climate, nutrients, water, and energy consumption, while IoT protocols controlled pumps, valves, heaters, and lamps. Experimental results showed a 20% reduction in water consumption, improved energy efficiency, and better resource management through real-time monitoring.

The use of software tools has also been studied in Agriculture 4.0. In 2016, Lopez-Riquelme et al. proposed using adequate resources for storing, managing, and processing information on precision agriculture. The authors demonstrated that using cloud services in the agronomic context is beneficial [17]. Big data applications in intelligent farming have also been used in the same context. Wolfert et al. conducted a literature review on this scheme. Despite these advances, several issues must be solved, such as the amount of data available for free for small producers and the security of the information [18,19].

As is well known, vegetable production is a fundamental and critical key because it is an elemental factor for the survival of humanity and the global economy. Thus, researchers have identified agricultural technology and precision farming as essential to ensure the current and future demand for food. Considering the above, several technologies have been proposed to improve its production. Moreover, recent advances in Agriculture 4.0 and other technologies, such as artificial intelligence and data science methodologies, facilitate understanding complex problems, such as predicting spinach growth. In 2018, Liakos et al. proposed an application of machine learning to manage a crop with predictions, disease detection, weed detection, and species recognition [20,21]. The authors employed various regression models, including linear regression, logistic regression, stepwise regression, and more complex algorithms such as ordinary least-squares regression, multivariate adaptive regression splines, multiple linear regression, and locally estimated scatterplot smoothing. The study demonstrated that these models effectively predicted key agricultural parameters, particularly in crop management, where Artificial Neural Networks (ANNs) were predominantly used for yield prediction and disease detection. The independent variables in the research encompassed environmental parameters such as soil moisture, temperature, and nutrient levels. The reported error rates varied across models and applications, with the ANN-based models achieving higher accuracy than traditional regression techniques, particularly in water and soil management tasks.

Other technologies, like Blockchain and Internet of Things systems, have been proposed by researchers and applied to Agriculture 4.0. For example, in 2020, Torkya et al. studied the importance of these technologies in the development of precision agriculture methods [22]. The authors reported integrating blockchain and the Internet of Things (IoT) in precision agriculture. The experimental results highlighted several obstacles to implementing blockchain-based IoT systems, including scalability, energy consumption, and interoperability challenges.

### 2.2. Data Science in Agriculture

Data science has been applied with several results and techniques; in 2012, Prasad applied artificial neural networks with transfer functions to retrieve spinach crop parameters, such as biomass, leaf area index, average plant height, and soil moisture content, for crop/vegetation monitoring [23]. Another approach was made in 2017 by Gent, who related the Relative Growth Rate of spinach with the environment and nutrition on growth using multiple linear regression [24]. Four years later, Torres used the modified partial least-squares regression (MPLS) technique to develop calibration models and applied near-infrared spectroscopy (NIRS) to monitor the growth of spinach plants in the field, aiming to enhance the understanding of agricultural practices and spinach harvest quality [25]. Concerning other subjects, applying mathematical models can help understand and predict the impact of fertilizers on plant growth under specific soil conditions. Descriptive statistics were applied to initiate data analysis, showcasing potential applications in optimizing agricultural practices and data-driven decision-making to enhance soil crop productivity. Krisnawati demonstrated this in 2021 [26]. In terms of deep learning as an algorithm based on a neural network that automatically selects data features, in 2021, Lu studied the classification of diseases in plant leaves using deep learning methods, specifically, convolutional neural networks (CNNs) [27]. Similarly, in the same year, Koyama utilized neural networks and convolutional neural networks in computer vision systems for classification in food quality assessment using the support vector machine model for both regression and classification, obtaining a sensory evaluation of spinach freshness [28]. Recently, in 2022, Islam and Sennam, in different studies, utilized the same deep learning technique through Convolutional Neural Networks (CNNs) to process images of various types of spinach and classify leaves among their different varieties with high accuracy and performance [29,30].

### 2.3. Assisted Agriculture Models

Agriculture models have been proposed to cover different aspects and features of spinach plants, including leaf growth, area size, and number of cells. In 1970, Chiara et al. studied the effects of optimal red–blue ratio in LED lighting for indoor horticulture. Results provided information about the growth patterns of spinach leaves, shedding light on the timing and distribution of cell division during their development [31]. The growth of spinach plants under controlled conditions in different soil types and the impact of nitrogen on it was also studied by Smolders et al. [32]. In that study, researchers found that the dry matter productivity of leaves decreases as the plants grow. This decrease was attributed to a reduction in the Net Assimilation Rate (NAR), which indicates that the rate of assimilation (conversion of light energy into biomass) per unit of leaf area decreases with plant growth.

Other experiments for understanding the effects of vacuum space on plant growth were conducted. In 1996, Iwabuchi et al. explored the impact of low pressure on spinach growth throughout the entire cultivation period [33]. The authors report examined spinach growth under hypobaric conditions in a controlled chamber with three pressure settings: a control (101 kPa total, 21 kPa $O_{2}$), low total pressure (25 kPa total, 21 kPa $O_{2}$), and low total and $O_{2}$ pressure (25 kPa total, 10 kPa $O_{2}$). ${\mathrm{CO}}_{2}$ was kept constant at 40 Pa. Growth parameters remained unaffected under 21 kPa $O_{2}$, but at 10 kPa $O_{2}$, shoot length and leaf area decreased while dry weight was unchanged. Net ${\mathrm{CO}}_{2}$ assimilation peaked in the low-total-pressure treatment but declined after 25 days in the ${\mathrm{low-O}}_{2}$ treatment. The findings suggest that spinach can grow under one-fourth of atmospheric pressure if $O_{2}$ and ${\mathrm{CO}}_{2}$ levels are maintained. Results revealed that it is possible to grow spinach plants in very low-pressure environments. Experiments showed that the growth rate measured in 10 days was similar to those obtained under atmospheric pressures.

Similarly, Kaminishi et al. investigated the seasonal changes in nitrate and oxalate concentrations in spinach [34]. The experiments were conducted under controlled laboratory conditions to determine the seasonal shift in nitrate and oxalate concentrations and the relationship between growth rate and concentration of nitrate and oxalate in spinach. The study applied changes in the experimental conditions, such as temperature, pressure, concentration, or exposure time, to assess their impact on different weather seasons. The results indicated that the growth rate of spinach cultivars is directly proportional to the nitrate and oxalate concentration. It was found that fast-growing cultivars contained high nitrate but low oxalate, whereas slow-growing cultivars contained low nitrate but high oxalate, regardless of the growing season.

In addition to the previous studies, the effect of chemical fertilizers on the growth rate of spinach was also studied. In 2010 and 2011, the National Research Center in Beheira, Egypt, investigated the impact of bio and chemical fertilizers on spinach plants’ growth, yield, and chemical properties [35]. The authors found that the synergistic application of a high-rate bio-fertilizer, specifically Piogen, in conjunction with a chemical fertilizer, is a productive approach for augmenting spinach plant growth, yield, and nutritional composition.

Following prior research, numerous models have been advanced to investigate alterations in spinach plants. Nevertheless, these endeavors have not comprehensively incorporated data about other aspects of the plant, including but not limited to height, number of stems, leaf count, and overall foliage area, along with comprehensive information regarding environmental conditions. Consequently, this paper proposes an integrated and comprehensive dataset designed for systematically examining spinach growth, aiming to address the existing gaps in understanding various plant parameters and environmental factors.

In the following section, Section 3, the general methodology used to acquire data from the spinach growing process is presented.

## 3. Materials and Methods

The spinach (*Spinacia oleracea* L.) plants were cultivated in a controlled indoor vertical farming system using a soil-based substrate enriched with vermicompost in a 3:1 ratio to optimize nutrient availability and soil structure. The substrate was prepared with the optimal values of nitrogen (N) at approximately 150 mg/kg, phosphorus (P) at 50 mg/kg, potassium (K) at 180 mg/kg, and magnesium (Mg) at 30 mg/kg. The soil pH was maintained within the optimal range of 5.7–6.8, supporting nutrient uptake and root development. Soil moisture levels were monitored and maintained between 60–75% field capacity, corresponding to a daily water deposition of approximately 200 mL per plant through an automated drip irrigation system. The experiment was conducted in five independent repetitions to ensure statistical robustness. The experimental period lasted 60 days for each pair of plants, and information from the key growth parameters was stored continuously. This methodological approach enabled the systematic observation of plant responses to environmental variables, fostering the development of robust predictive models for spinach growth dynamics within vertical farming systems.

**Figure 1:**
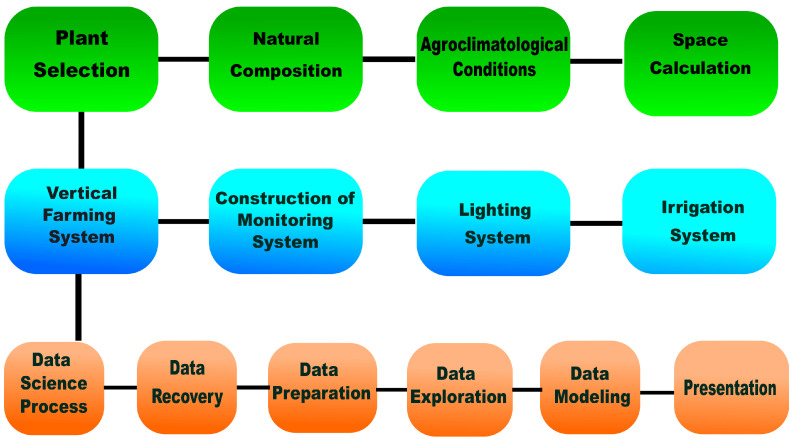
Proposed work block diagram for spinach growth modeling.

The block diagram of Figure 1 represents the proposed stream-lined methodology forwarded to study spinach growth. The pipeline steps involved in the workflow are as follows: (i) plant selection criteria, (ii) vertical farming system, and (iii) data science process. The stepwise work progress is described below.

**1. Plant Selection Criteria Considering Energy Content and Growth Conditions:** The chosen spinach crop is (*Spinacia oleracea* L.), renowned for yielding 31 Kcal per 100 g of its edible portion. Spinach (*Spinacia oleracea* L.) was selected as the study material due to its high nutritional value, adaptability to controlled environments, and efficient resource utilization. This leafy vegetable is widely recognized for its rapid growth cycle and suitability for precision agriculture techniques. It is an ideal candidate for research on optimizing growth conditions using sensor-based monitoring and Agriculture 4.0 technologies. The chosen variety, Viroflay, was selected based on its superior agronomic and nutritional characteristics. This cultivar is renowned for its high biomass productivity, averaging 22 plants/m² with a mean harvestable plant weight of 355 grams.

Additionally, Viroflay exhibits a high seed germination rate, a faster growth cycle than other spinach varieties, and low water and nutrient requirements, making it highly efficient for resource-conscious agricultural systems, as reported by Grevsen et al. [36]. Furthermore, it has been documented to perform well across diverse environmental conditions without requiring extensive agronomic interventions. From a nutritional perspective, Viroflay provides 31 kcal per 100 g of edible portion and is an excellent source of essential micronutrients, including Vitamins A and C, folic acid, iron, magnesium, phosphorus, potassium, selenium, calcium, and dietary fiber. These attributes contribute to its prominence as a high-value crop for industrial and scientific applications. Additionally, the optimal growth conditions for Viroflay include a temperature range of 14–18°C and relative humidity of approximately 75%, making it well suited for controlled-environment agriculture. This variety has also been associated with high chlorophyll content, large leaf size, and dark green foliage, which improve its suitability for industrial processing and precision agriculture studies. By selecting Viroflay, this research aims to leverage its agronomic advantages to develop advanced methodologies for modeling growth.

**2. Infrastructure to Facilitate Seed Planting, Germination, Growth, and Harvest in a Controlled Growth Environment:** This study clarifies the design and deployment of an infrastructure tailored for a monitored vertical farming system. The system is engineered to streamline the complete cultivation lifecycle, encompassing activities such as planting, germination, growth, and harvest, all meticulously managed within a controlled growth environment. Central to this infrastructure is incorporating LED lighting, providing a wide-spectrum illumination devoid of heat generation and enabling the selective utilization of diverse wavelengths.

Growing *Spinacia oleracea* L. demands illumination with a spectral composition that influences the physiological processes. This study employed a customized LED lighting system to enhance spinach growth by providing a balanced combination of red (630–660 nm) and blue (450–470 nm) wavelengths, essential for maximizing photosynthetic activity and regulating plant morphology. Red light activates phytochrome signaling pathways, promoting stem elongation, chlorophyll production, and biomass accumulation. It also significantly stimulates the synthesis of gibberellins and cytokinins, hormones that regulate cell division and leaf expansion. On the other hand, it was reported by Matzuda et al. [37] that blue light is essential for chloroplast development, enhancing photosynthetic efficiency and encouraging compact, structurally robust growth.

Additionally, blue light influences auxin production, affecting root formation and phototropic responses while preventing excessive stem elongation, resulting in more compact plants. By integrating LED-based spectral modulation within the monitored vertical farming system used in this study, it is possible to control light exposure precisely, optimizing key hormonal pathways to improve spinach growth. This approach enhances morphological characteristics and increases nutrient content, making it a critical element in precision agriculture and high-density cultivation systems.

It has been empirically validated that the combination of red and blue light is highly effective for cultivating vegetables [7]. The judicious manipulation of the provided light source enhances crop growth and saves energy. The selective variation in the light spectrum supports optimal plant development and contributes to sustainable energy usage within the vertical farming system.

**3. Data Science Process for Extraction, Exploratory Data Analysis, and Processing of Meaningful Information to Model Plant Growth:** The development of data science applications demands specific stages that involve the extraction, cleaning, exploring, and processing of the data. Thus, the proposed model takes into account the following stages:

- Data Retrieval—This stage involves accessing and extracting the generated information while selecting appropriate data structure tools. A methodology based on data transmission and storage infrastructure was used using the MQTT protocol for IoT applications.
- Data Preparation—We must eliminate elements irrelevant or significant to our objectives from the generated data. Information was prepared and considered using different time scales from the data stored for each physical variable studied. For example, we have the temperature measurement inside the box (with a sampling frequency of every 6 s), while the images are acquired every 10 min. Consequently, different temporal representation scales are selected for the study for each type of sensor, which implies having different samplings in the spinach plant database.
- Data Exploration—This phase entails exploring data to identify distinctive features using data visualization methods. All data were graphed to identify trends in the behavior of the variables and their evolution over time. Similarly, other analyses were carried out on the data trend considering minimum, average, and maximum values, compared with control references used in the state of the art.
- Data Modeling—This stage aims to discover or generate reference, prediction, or classification models. Artificial intelligence algorithms were utilized to model and forecast the behavior of preprocessed data concerning variables earmarked for predicting spinach plant growth. Specifically, these algorithms fall into two categories: digital image processing and supervised learning regression algorithms.
- Presentation and Automation—The final stage involves presenting the conclusions drawn from the results. This may also include the automation of processes to streamline ongoing analyses. The obtained data signify a shift in the transformation of the representation space of physical variables governing the growth of spinach plants within a controlled enclosed setting. It is noteworthy that precision agriculture tools facilitate the identification of crucial factors influencing spinach development under optimal growth conditions.

The systematic execution of these data science stages ensures a comprehensive approach to extracting valuable insights into plant growth patterns. This process is fundamental for advancing our understanding of the factors influencing plant development and enhancing agricultural practices. However, obtaining precise measurements of leaf and stem characteristics in spinach plants presents significant challenges due to their intricate structures and delicate nature. Consequently, manual measurements were conducted to ensure accuracy and reliability in data collection.

### 3.1. Plant Selection

**Figure 2:**
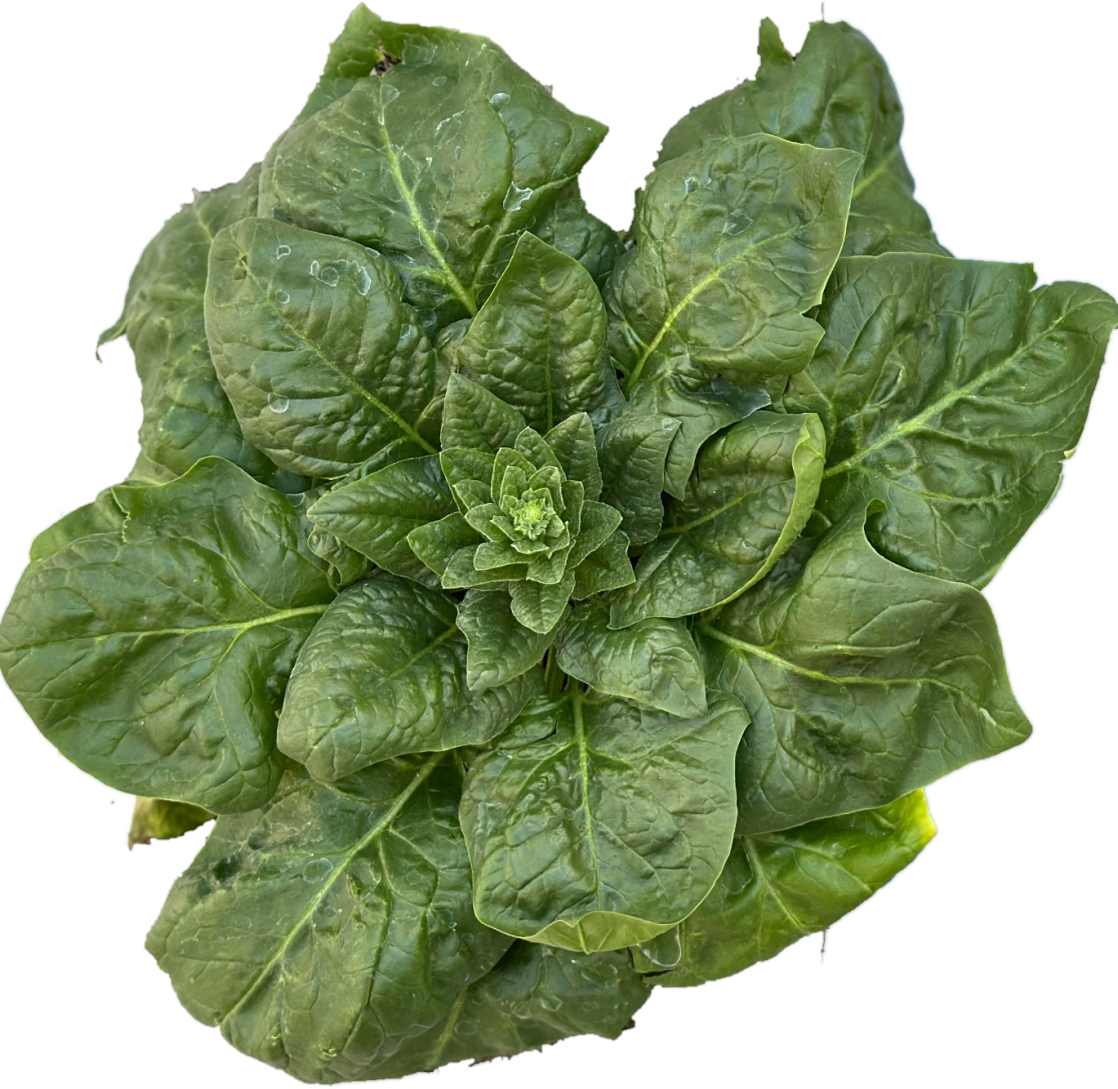
Spinach (*Spinacia oleracea* L.).

The selection of cultivable plants focuses on (*Spinacia oleracea* L.) due to its significant contribution in nutrient content and energy provision, amounting to 31 Kcal per 100 g (Figure 2). Spinach production predominantly centers on its green leaves, with lighting and temperature as pivotal factors influencing plant development. Therefore, it is imperative to maintain appropriate photoperiods with temperatures exceeding 15 °C. Optimal leaf development occurs within the temperature range of 14 to 18 °C, and illumination periods of 1500 to 1700 h annually. Some of the most important parameters associated with spinach cultivation are presented in Table 1.

In addition, spinach is a nutrient-dense vegetable, rich in essential vitamins and minerals. It is an excellent source of vitamin K, vitamin A, folate, and iron, among others, making it a valuable addition to a balanced diet. Moreover, its low-calorie content makes it an ideal choice for individuals aiming to manage their weight while ensuring adequate nutrient intake. Incorporating spinach into a daily diet offers numerous health benefits, including improved cardiovascular health, enhanced digestion, and strengthened immune function. With its versatile culinary applications and impressive nutritional profile, spinach remains a staple in various cuisines worldwide.

### 3.2. Agriculture System

**Figure 3:**
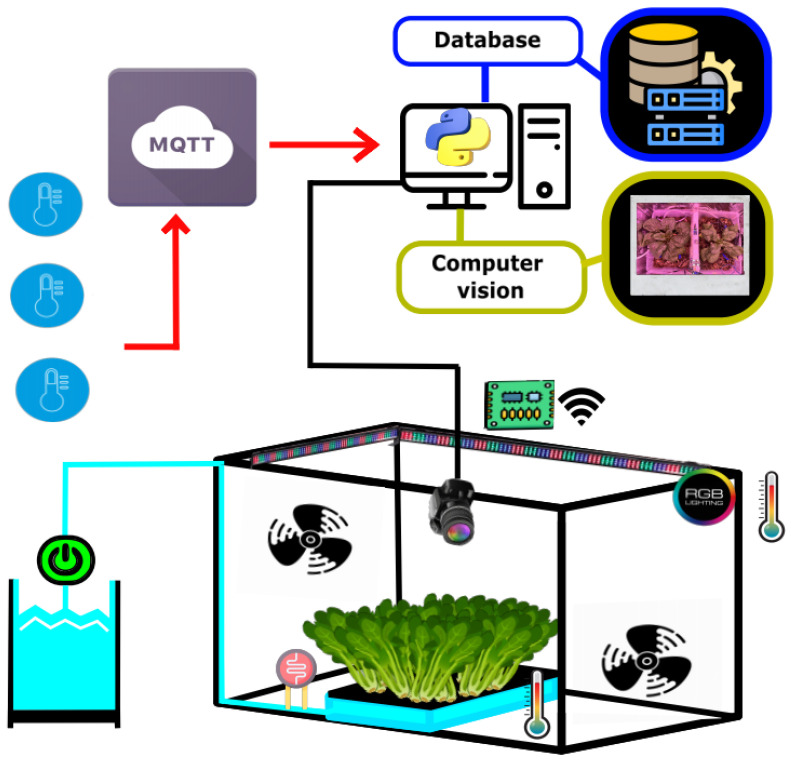
Enclosed agricultural system with IoT sensor–broker architecture.

**Figure 4:**
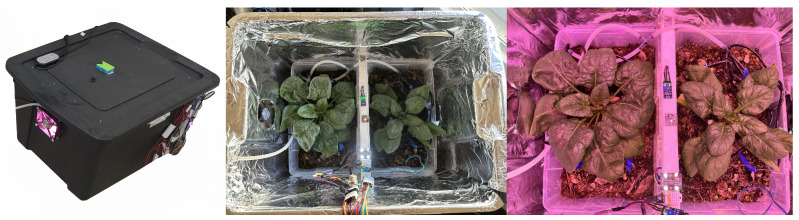
Prototype for the enclosed precision agriculture system. The central and right images showcase a top view of the cultivation area featuring two spinach plants: one under natural lighting in the central section and the other illuminated by LED-controlled lighting on the right side. Additionally, visible are the temperature, humidity, and light intensity sensors, which are essential components of the developed IoT system.

The planting space is a closed space of 60 × 41 × 32 cm^3^ made of multipurpose polypropylene to contain a substrate of 15 × 30 × 15 cm^3^. The substrate was enriched with vermicompost, a nutrient-rich organic amendment derived from earthworm activity, at a ratio of 1:3 with soil. Vermicompost enhances soil structure by improving aeration, porosity, and water retention while providing essential plant nutrients in bioavailable forms, such as nitrates, exchangeable phosphorus, and soluble potassium [38]. The spinach (*Spinacia oleracea* L.) maximizes its production in environmental conditions of 75% relative humidity and temperature between 14 and 18 °C in 12 h photoperiods. Figure 3 and Figure 4 illustrate the complete agriculture system.

#### 3.2.1. Lighting System

A layer of aluminum foil is applied to reflect the light beams the plants use, ensuring uniform light distribution within the system. This also helps optimize the reach of the light emitted by LED strips for plant cultivation.

Particularly, the lighting system uses four units of 192 80 W full-spectrum LEDs with automatic on/off timers, including 84 red LEDs (630 nm), 44 blue LEDs (460 nm), and 16 yellow LEDs (3000 k). The programming of the system included 12 h photoperiods, during which 100% brightness was utilized with the concurrent activation of yellow, blue, and red lights, considering agroclimatic conditions. The incorporation of yellow lights in the system facilitates the introduction of a minor proportion of green light (20%) to enhance plant growth [39].

#### 3.2.2. Irrigation System

An automatic drip irrigation system was implemented to deliver 200 mL of potable water per day in two events. The amount of liquid was estimated considering the volume of the plant pot.

#### 3.2.3. Temperature Control System

The temperature control system employed air cooling driven by two 4-inch general-purpose fans. Following agroclimatic recommendations, the temperature threshold was set at 18 °C.

### 3.3. Data Acquisition System

The instrumentation system is comprised of two distinct components. Firstly, a sensor system was deployed to monitor various environmental variables, gathering data at 10 s intervals. These variables included temperature and humidity levels within the enclosed plastic box, substrate humidity, room temperature and humidity, intensities of red, green, blue, and white illumination, and the photoperiod of the illumination cycle.

Moreover, an image sensor system was utilized to capture visual data illustrating the temporal evolution of plant growth, capturing frames at 10 min intervals.

**Figure 5:**
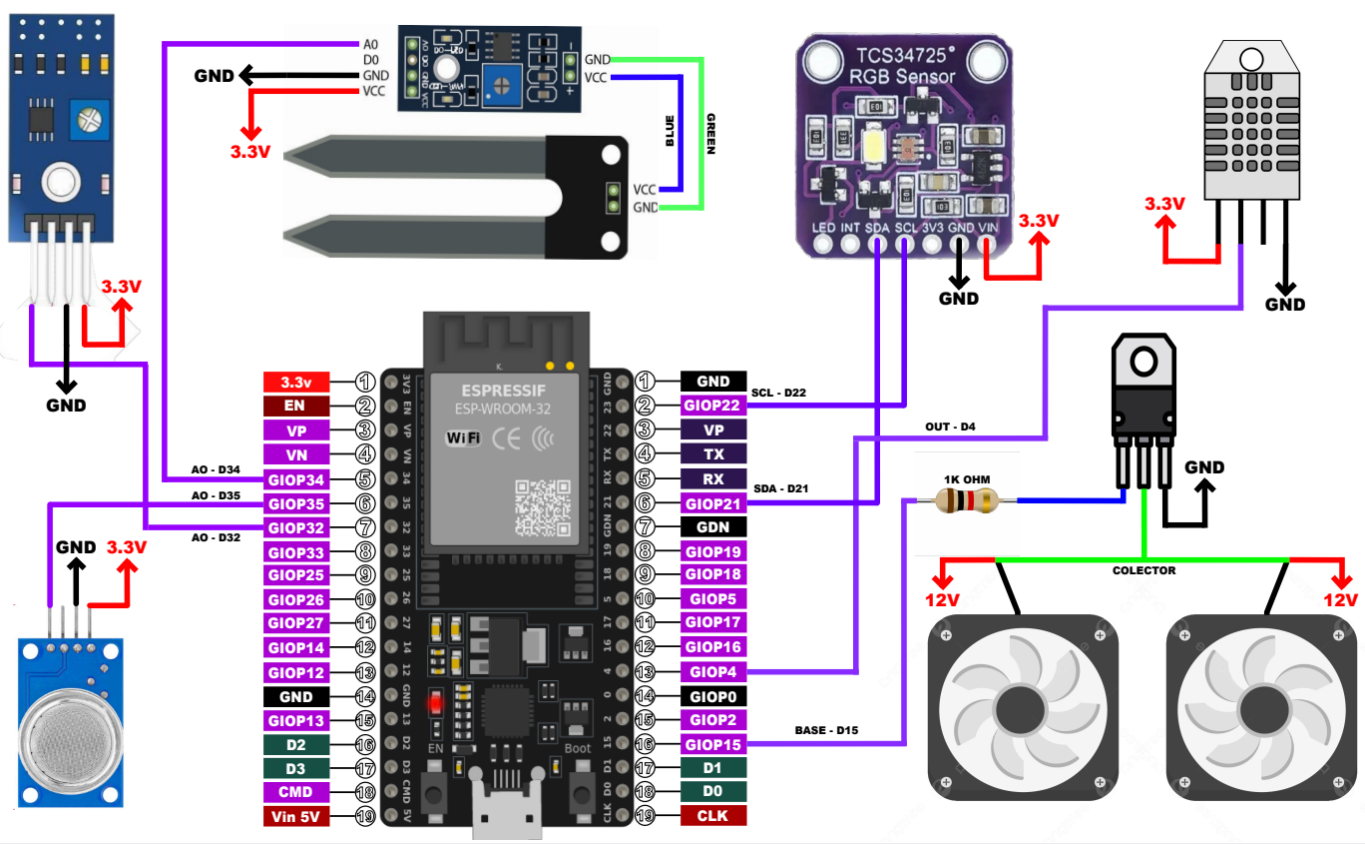
Block diagram of the electronic data acquisition system.

The electronic data acquisition system utilized an ESP32 process board as its core component, providing the capability to acquire, process, and transmit data collected from the sensors to the IoT broker. Additionally, a power interface incorporating a transistor was integrated to regulate the operation of fans responsible for altering the airflow to and from the external environment. The schematic diagram illustrating the electrical connections of the proposed system is depicted in the accompanying Figure 5.

The firmware of the electronic system was developed in micropython in the Thonny-IDE development environment. The TCS34725 color sensor communicates using the I2C communication protocol, facilitating seamless interfacing with the ESP32. Moreover, alongside the I2C protocol, the TCS34725 features an interrupt signal output capability to read the intensity values in the red, green, and blue wavelengths.

#### 3.3.1. Sensor and Actuator System

The study employed a suite of commercially available sensors, as detailed in Table 2. Temperature and humidity were measured using the DHT22 sensor, offering 8-bit resolution within a 0–256 range at a sampling rate of 6 s. The substrate temperature was monitored using the MNS2-9 sensor with a 12-bit resolution. In contrast, room temperature and humidity were recorded using the KSUM5 sensor, operating at a 10-bit resolution with sampling intervals of 6 and 10 s, respectively. Soil moisture content was measured using the FC28YL38 sensor, which provided a 12-bit resolution. Light intensity across red, green, blue, and white spectra was assessed using the TCS34725 sensor, which features a 16-bit resolution and a sampling rate of 6 s. Additionally, energy consumption monitoring was performed using the MNS29IN sensor with a 12-bit resolution and a 60-s sampling interval, while visual data for plant growth analysis were captured using the C920LOGI camera at 1920 × 1080 resolution.

To ensure measurement reliability, all sensors underwent a rigorous calibration process. The DHT22 humidity and temperature sensors were factory-calibrated, with correction coefficients stored internally. For additional validation, readings were cross-compared against a high-precision reference instrument in a controlled environment. The TCS34725 color sensor, which operates on the I2C protocol, was calibrated by exposing it to known light intensity values across different spectral ranges and adjusting its internal gain settings accordingly. Soil moisture sensors were calibrated using reference soil samples of known moisture content, ensuring accurate substrate humidity readings.

Overall, the selection and calibration of sensors aimed to optimize measurement accuracy while maintaining a cost-effective and scalable system for precision agriculture applications.

Table 2 presents the technical specifications of all sensors employed in data acquisition for the enclosed spinach cultivation system. These sensors capture various parameters, including temperature, humidity, substrate conditions, light intensity, and energy consumption. Each sensor model is defined by its resolution, range, and sampling rate, furnishing indispensable metrics for precise data acquisition and analysis. The information these sensors provide facilitates thorough surveillance of environmental factors crucial for optimizing spinach cultivation in the closed-controlled agriculture system. In addition, this data acquisition infrastructure serves as a fundamental element within the proposed methodology, streamlining the extraction of features and the subsequent modeling of spinach growth dynamics from data.

#### 3.3.2. Dataset

The data acquisition system meticulously selected and processed information for analytical purposes. The dataset covers 60 days from the initial sowing of spinach seeds to the completion of their growth.

**Figure 6:**
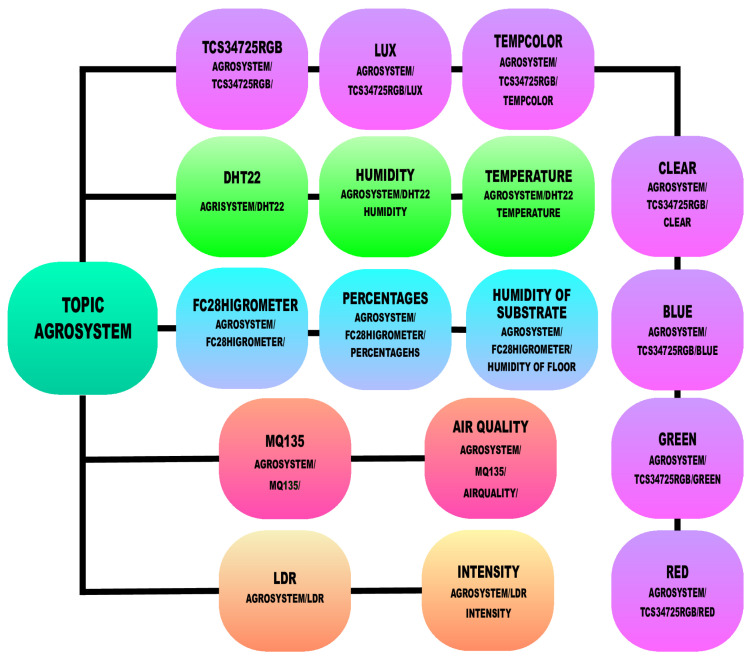
Structure of data topics within IoT Broker.

This dataset was constructed utilizing data obtained from environmental parameters and imagery. A set of 13 sensors was employed to digitize the environmental conditions surrounding the spinach plants. Furthermore, the vision system captured a total of 6288 images. Figure 6 illustrates the structure of the sensor information stored in the IoT Eclipse Mosquitto, an open-source (EPL/EDL licensed) message broker that implements the MQTT protocol. Four main topics were created to collect information on the environmental variables. The inside and outside sensors of the same type were subscripted to the same IoT Topic.

**Figure 7:**
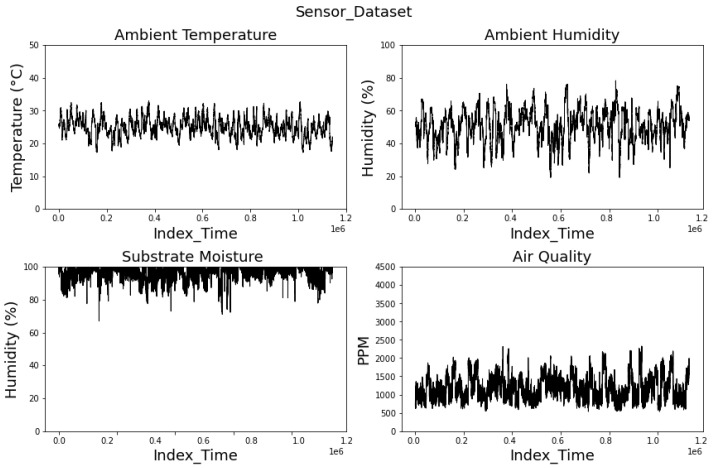
Environmental data within the enclosed agricultural system.

The dataset consists of two primary components: environmental variables and images. The environmental variables include discrete data representing various physical magnitudes, with more than 21 million data points collected. Figure 7 presents the recorded environmental parameters, including ambient temperature, ambient humidity, substrate moisture, and air quality. The top-left plot depicts ambient temperature (°C), exhibiting fluctuations within a relatively stable range, with an average temperature of 25 °C. The top-right plot displays ambient humidity (%), demonstrating dynamic variations over time. The bottom-left plot represents substrate moisture (%), which remains high throughout most spinach growth cycles. Lastly, the bottom-right plot illustrates air quality in terms of parts per million (PPM), showing noticeable fluctuations. The average air quality measurement was 1000 PPM, indicating favorable conditions for spinach cultivation.

These sensors provide detailed insights into the environmental conditions surrounding the spinach cultivation. On the other hand, the image data captured to monitor the growth progression of spinach plants was also stored. Both data structures offer crucial information for understanding and optimizing the spinach growing system under a controlled environment.

**Figure 8:**
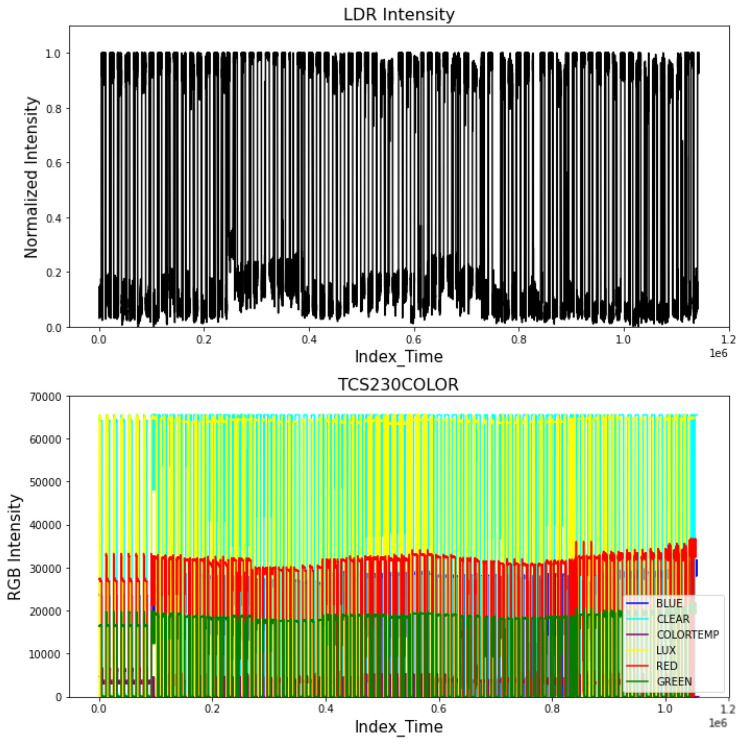
Illumination pattern depicting the historical growth cycle of a spinach plant.

The light intensity and spectral data for red, green, and blue wavelengths were measured throughout the growth period. This information is valuable for monitoring the radiated energy projected onto the spinach plant. Figure 8 shows the average light intensity in the upper section, with intervals indicating when the light was on or off. The lower plot presents the spectral energy centered on red, green, and blue wavelengths. The total energy radiated during the entire growth process can also be derived from the normalized intensity. Historical data can also be utilized to detect system failures caused by electrical issues in the enclosed agriculture system. However, this information is not considered when modeling the growth process. Nevertheless, these data could help monitor the correct operation of the agriculture systems and ensure the proper amount of energy radiated each day.

## 4. Modelling Spinach Growing

**Figure 9:**
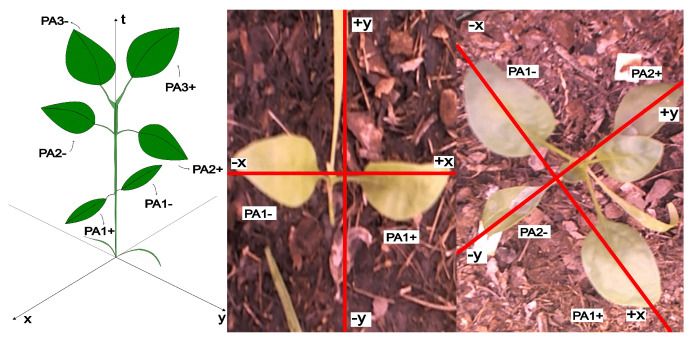
Leaf reference coordinate identification system.

Like other similar plants, spinach holds roots, stems, branches, and leaves. However, no specific method exists for labeling one of the elements under study using a high-precision agricultural system. We propose using the following strategy to identify each spinach leaf. First, all leaves grow in pairs, so we numbered them according to their order of appearance. Then, the notation for leaf pairs is PlantID, PairID, and the Orientation symbol is positive (+) or negative (−). We use a Cartesian coordinate system to identify a given pair of leaves; see Figure 9.

### 4.1. Spinach Growing Features Extraction

**Figure 10:**
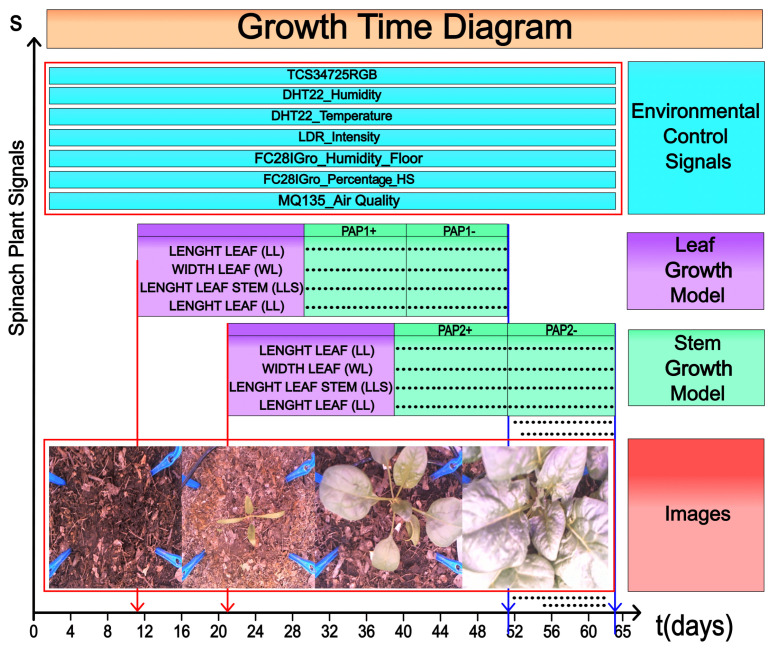
Spinach growth time diagram with all information sensors and variables extracted.

The combination of all variables and features extracted from spinach plants during the growing time is presented in a single-time diagram, as illustrated in Figure 10. The X and Y axes represent the time in days and all signals acquired and extracted from the plant. Notably, the Growth Time Diagram (GTD) is divided into three types of signals: Environmental Control Signals (ECSs), leaf and stem features, and images acquired from the zenith angle of the plant.

The ECS represents the environmental information of the plant where it was growing. Each sensor, TCS34725RGB, DHT22 (humidity and temperature), LDR Intensity, FC28IGro (humidity), and MQ135 (air quality), gathers information from the enclosed agriculture system.

Additionally, the leaf length, stem width, and stem length were manually measured using a vernier caliper due to the complexity of morphology and multiple occlusions among the leaves of the plants, which hindered the application of computer vision techniques.

Measurements were conducted daily at noon, beginning once the respective leaf became visible. The P1 leaf was monitored from day 1 to day 42, while P2 was measured from day 4 to day 49. Similarly, P3 was assessed from day 9 to day 47, P4 from day 12 to day 47, P5 from day 20 to day 49, P6 from day 24 to day 47, P7 from day 31 to day 50, P8 from day 34 to day 53, P9 from day 37 to day 57, P10 from day 42 to day 58, P11 from day 45 to day 58, P12 from day 49 to day 58, and P13 from day 49 to day 59. To model the growth of each leaf and stem with high accuracy, the polynomial regression fit only considered the interval of time where the pair was visible rather than the total length of the experiment with the germination period. The above reduces the interval where the leaf or stem is not visible.

A critical aspect of the GTD is that all sensors hold different spatial information, acquisition time, and resolution. However, it is possible to generalize that any features measured in a plant could be grouped into the three main categories proposed in this work.

**Figure 11:**
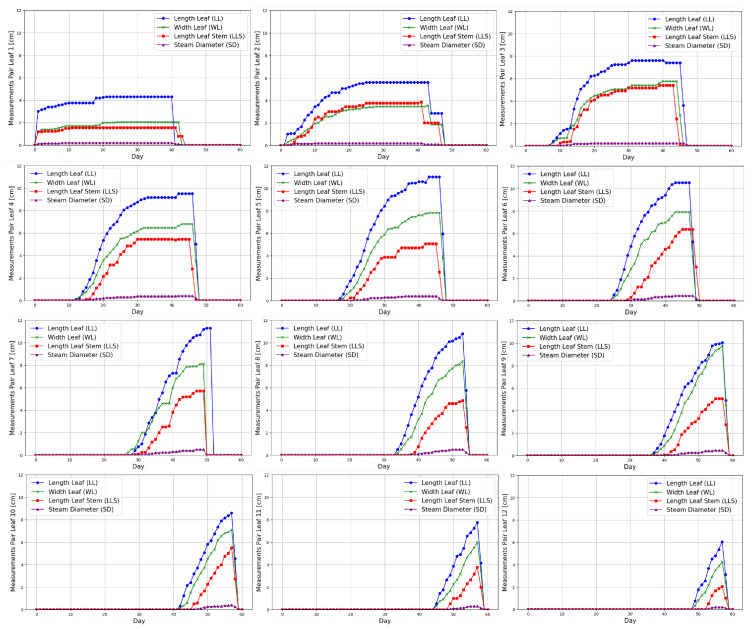
Progression of spinach leaf growth over 60 days, featuring key metrics such as leaf length, width, leaf stem length, and stem diameter.

Figure 11 illustrates the growth progression of a spinach plant leaf over 61 days, showcasing key metrics such as leaf length, width, leaf stem length, and stem diameter. Initially, the measurements are pretty low (small), indicating the early stages of development. As time progresses, all metrics show a noticeable increase, particularly in leaf length and width, reflecting the vigorous growth of the plant. The data highlight distinct growth phases, with significant size increases observed after specific periods, suggesting optimal conditions for development. The stabilization of measurements towards the end indicates the plant is reaching maturity, where growth rates may slow down. Overall, this figure effectively captures the dynamic growth pattern of spinach leaves, providing insights into their development under suitable environmental conditions.

### 4.2. Spinach Growing Regression Models

A set of regression models is proposed to model the growth of the spinach plant. Each leaf grows at a different rate and has a unique lifecycle, requiring multiple regression models to capture the growth dynamics of varying leaf pairs. Figure 11 visually represents these models for each leaf pair, showing the variation in polynomial trends over time. Each polynomial regression model is tailored to a specific pair of leaves, reflecting the distinct growth patterns, and the differences in model shapes emphasize the variability in growth behavior across the plant lifecycle.

The predictive model developed in this study was constructed using machine learning techniques to analyze and optimize plant growth conditions in a controlled environment. The dependent variables included temperature, relative humidity, soil moisture, light intensity, and plant growth parameters (e.g., height and leaf expansion). In contrast, the independent variable was the time (days after planting), which allowed for tracking growth trends over time. To build the model, the dataset was split into training (70%), validation (15%), and test (15%) subsets to ensure model generalization and prevent overfitting. The training set was used to adjust model parameters, the validation set optimized hyperparameters, and the test set assessed the final model performance. Regression techniques, including polynomial and nonlinear models, were explored, with the best-performing model selected based on statistical metrics such as adjusted coefficient of determination, residual standard deviation, and Akaike Information Criterion. Additionally, cross-validation was employed to enhance robustness, and model assumptions were verified through residual analysis.

This polynomial regression model aims to fit a curve to data representing stem diameter growth over 60 days. The code uses the Polynomial Features and LinearRegression classes from the Sklearn library to create and evaluate polynomial regression models of different degrees.

The process begins by defining the data, where X represents the days (ranging from 0 to 60), and y is the measured stem diameter at each corresponding day. The stem diameter data starts at zero, with growth observed later in the period, increasing and stabilizing.

The code attempts to fit polynomial models of varying degrees (from one to nine) to the data and calculates the mean squared error (MSE) for each model to determine how well it fits the data. The model with the lowest MSE is the best fit, indicating the degree of the polynomial that minimizes prediction error.

The coefficients of the best polynomial model are displayed, and the corresponding regression curve is plotted alongside the original data. This visual comparison shows how closely the model matches the stem diameter growth over time, providing an intuitive way to assess its effectiveness. In this case, the model helps capture the nonlinear growth patterns of the stem over 60 days.

In Table 3, Table 4, Table 5 and Table 6, the first column represents the order of the polynomial learned (Model), while P1 to P12 represents the leaf pair studied. The values provide a quantitative assessment of model accuracy, with lower MSEs indicating a better fit to observed growth patterns. The variability in MSE across leaf pairs suggests differences in growth trajectories influenced by environmental factors and biological variability. Leaf width models tend to have lower MSE values than leaf length models. In contrast, leaf length and stem length models show higher variability, probably due to environmental factors and the physiological adaptation of plants.

## 5. Experimental Results

The growth of spinach leaves and the use of polynomial regression modeling to predict essential parameters of leaf development were organized by analyzing polynomial coefficients and mean square errors (MSEs) across different growth dimensions: Leaf Pair Length, Leaf Pair Width, and Leaf Stem Length.

The evaluation of the proposed models is based on mean squared error, a metric particularly well known for polynomial regression. MSE measures the squared differences between measured and predicted values, making it a practical choice for modeling leaf length, width, and stem length. Alternative metrics, including Relative Approximation Error (RAE), Mean Absolute Error, and Mean Absolute Percentage Error, along with Maximum Error (MAX) and Maximum Percentage Error (MAXP), were not considered due to the stable nature of the spinach growth parameters and the study’s focus on minimizing overall prediction error rather than relative or absolute deviations. Although RAE and MAPE are advantageous for datasets with wide-ranging values, and MAX and MAXP help identify extreme deviations, these metrics do not offer additional benefits over MSE in this context. Additionally, MAE’s lower sensitivity to large errors may underestimate significant variations in polynomial models. MSE enhances computational efficiency, maintains consistency in model evaluation, and effectively accounts for leaves and stems in plant growth.

The polynomial models for the Leaf Length Pair dimension (Table 7) include coefficients up to the ninth degree, aiming to capture nuanced variations in leaf length as growth progresses. The mean square error (MSE) values, specific to each leaf pair, provide insights into model precision. Particularly, Pair 2 has one of the lowest MSEs at 0.158, while Pair 5 shows the highest at 1.2153, suggesting variability in model fit across pairs. Similarly, the Width Leaf Pair models (Table 8) use ninth-degree polynomials with lower MSE values than those for length. Pair 12 achieves the lowest MSE at 0.0741, while Pair 7 has the highest at 0.822, indicating a more consistent fit for width, which may imply more stable lateral growth patterns in leaf morphology.

While developing the analyses (Stem Length and comparative model performance), the polynomial models for Leaf Stem Length pairs (Table 9) also feature terms up to the ninth degree. The MSE values suggest a relatively good fit for stem length, with the lowest MSE of 0.0312 for Pair 1, indicating high predictive accuracy. However, specific pairs, like Pair 7 with an MSE of 0.3907, reveal that the model may not fully capture sporadic changes in stem growth. Across dimensions, polynomial regression appears particularly effective for Width Leaf Pair and Stem Length, where MSE values are generally lower, suggesting more predictable growth (Table 10). In contrast, the higher MSEs for Length Leaf Pair indicate more significant variability, potentially attributable to complex factors influencing biological growth. Future improvements might explore alternative models or fitting techniques to reduce MSE further, enhancing predictive capabilities and supporting agricultural optimization.

In addition, experimental results in Table 7, Table 8, Table 9 and Table 10, particularly the observed mean square error (MSE) values, do not exhibit the typical characteristics of extreme overfitting, where the training error is significantly lower than the expected generalization error. The MSE values across different leaf growth parameters (length, width, and stem length) maintain variability and are not excessively low, indicating that the models capture patterns rather than memorizing the training data. Furthermore, the fitting error observed across various polynomial degrees suggests that the model does not entirely lose generalization capability, as expected in severe overfitting cases. The fluctuations in MSE values across different leaf pairs and stem lengths provide further evidence that the model encounters natural biological variability rather than artificially minimizing error through excessive complexity. While regularization techniques can further refine model performance, the current polynomial regression models effectively balance fit and generalization, as demonstrated by the distributed error metrics.

## 6. Discussion

The results obtained from the experiments provide information about the influence of environmental variables on spinach growth within an enclosed precision agriculture framework. The continuous acquisition of ambient temperature, humidity, substrate moisture, and air quality data enabled a comprehensive evaluation of microclimatic conditions affecting plant development. The ambient temperature data showed that the optimal range for spinach cultivation must be around 25 °C. On the other hand, we found that ambient humidity was significantly high, which affects transpiration rates. The substrate moisture levels remained consistently high, which means easy hydration and nutrient absorption for the spinach plants. The air quality analysis, expressed in parts per million (PPM), revealed an average of 1000 PPM, within the optimal threshold for spinach growth.

Additionally, the dataset opens the understanding of growth patterns for each leaf pair and the whole spinach plant. Polynomial models applied up to the ninth degree effectively captured complex growth patterns, with mean squared error (MSE) values serving as indicators of model accuracy. Among the leaf length models, Pair 2 exhibited the lowest MSE (0.158), whereas Pair 5 recorded the highest MSE (1.2153). Similarly, for leaf width, Pair 12 achieved the lowest MSE (0.0741), while Pair 7 showed the highest (0.822). Pair 1 demonstrated the highest predictive accuracy for leaf stem length with an MSE of 0.0312. In contrast, Pair 7 exhibited the most significant variability with an MSE of 0.3907.

It is notable that results obtained in this study demonstrate the superior predictive capability of higher-order polynomial regression models in describing spinach growth patterns. Nevertheless, in 2016, Muianga et al. [40] and Ribeiro et al. [41] used Logistic and Gompertz models to describe the growth of pepper plants using fewer than 20 observations collected over 100 days of experiments. In contrast, the present study uses a larger dataset, allowing a better data-driven approach to growth modeling. The increased sample rate of the dataset and the use of higher-order polynomials provide improved resolution in capturing the non-linear growth dynamics of plants. Thus, the efficiency of the regression model is demonstrated by its ability to accurately predict growth trends, as evidenced by the low MSE values in specific leaf pairs, indicating high predictive precision.

Comparing the current results against those presented by Jane et al. highlights an inverse relationship between MSE and $R^{2}$, as models with lower MSE values exhibited higher $R_{adj}^{2}$, expressing improved data fitting. Moreover, the standard deviation of residuals (RSD) followed the same trend as MSE, reinforcing the accuracy of models with lower prediction errors. The AIC values provided additional insight into model selection by balancing goodness-of-fit with model complexity, penalizing overparameterized models despite achieving lower MSE. While polynomial regression models demonstrated low MSE values, the increasing complexity of higher-degree polynomials resulted in marginal improvements. These findings present the necessity of integrating multiple evaluation metrics to ensure the development of robust and generalizable plant growth models beyond simple error minimization.

In general terms, the MSE values obtained in this study for polynomial regression models demonstrate their effectiveness in capturing spinach growth dynamics. However, compared to advanced deep learning approaches, such as the Long Short-Term Memory (LSTM) networks utilized by Alhnaity et al. to model tomato yield, the observed differences highlight the potential benefits of incorporating deep learning techniques [42]. The authors applied LSTM models to predict plant growth and yield in greenhouse environments, achieving MSE values as low as 0.002 for tomato yield prediction and 0.001 for Ficus stem diameter variation. The above values are lower than those obtained using polynomial regression, suggesting that deep learning models may offer superior predictive capabilities by considering nonlinear growth patterns. Despite the higher accuracy of LSTM networks, polynomial regression is computationally efficient and interpretable, making it suitable for applications where model transparency and ease of implementation are critical.

A contributing aspect of the study is that polynomial regression models in the enclosed system represent a significant advancement in Agriculture 4.0 technologies, enabling real-time growth monitoring and precise predictive modeling of spinach under controlled conditions. Moreover, the accurate models can capture nonlinear growth dynamics for analyzing plant development. The use of machine learning for anomaly automatic detection contributes significantly to developing a system that identifies and responds to deviations from expected growth trajectories, ensuring optimal physiological growth of the plant.

## 7. Conclusions and Future Perspectives

This study demonstrates the efficacy of Agriculture 4.0 technologies in modeling the growth dynamics of Viroflay spinach (*Spinacia oleracea* L.) through polynomial regression. The study successfully captured the nonlinear growth patterns of individual leaf pairs, stems, and other plant structures over 60 days by employing regression models up to the ninth degree. The mean squared error (MSE) analysis revealed significant variations in predictive accuracy, with specific leaf pairs exhibiting stable, highly predictable growth trajectories. In contrast, others—particularly between Pairs 2 and 9—displayed more significant variability.

A comparative quantitative analysis of different regression models indicated that a ninth-degree polynomial function provided the most accurate individual leaf pair growth predictions. Although polynomial regression has proven effective in modeling spinach growth, a key limitation of this study is its exclusive dependence on this technique for predictive analysis. While polynomial models offer significant insights into plant development trends, they may not adequately represent the intricate and nonlinear relationships between environmental factors and plant physiological responses. However, future research will explore advanced machine-learning approaches to improve prediction accuracy and scalability, including deep learning and hybrid modeling techniques. Additionally, further experiments will incorporate physiological parameters such as chlorophyll fluorescence and conductance, along with environmental control variations, including temperature modulation, water availability, and adjustments in light intensity and photoperiod cycles. On the other hand, improving the enclosed agricultural system by integrating additional sensors to capture critical plant growth parameters, such as substrate pH and photosynthesis efficiency from leaf surfaces. Also, modifications to the illumination conditions—including intensity adjustments, spectral wavelength variations, and irrigation cycle optimization—will be explored to develop novel growth models for spinach under diverse environmental stressors. A new data integration framework will merge sensor-derived parameters and image-based features into a unified dataset, facilitating the application of deep learning techniques to model plant growth. Furthermore, a blockchain-based data representation framework will enable secure, standardized monitoring, control, and forecasting of spinach growth across varying environmental conditions.

In summary, the growth models derived for each leaf pair and stem serve as a fundamental mathematical framework for monitoring and regulating plant development within a controlled environment agriculture system. In future experiments, the agricultural monitoring system will automatically detect any deviation from the reference growth model, significantly contributing to the advancement of autonomous plant growth management.

## Figures and Tables

**Table 1:** Agroclimatic conditions suitable for spinach cultivation.

Agroclimatic Conditions	
Altitude	1430 to 2800 m above sea level (m.a.s.l)
Temperature	Germination 7–23 °C, Growth 15–25 °C.
Relative Humidity	60–75%
Water Requirement	800–1600 mm/year
pH Range	Moderately acidic values 5.7–6.8
Illumination	12 h of light

**Table 2:** Technical specifications of sensors for the enclosed agricultural system.

Variable	Model	Resolution (Bits)	Range	Sample Rate(s)
Temp.	DHT22	8	0–256	6
Humidity	DHT22	8	0–256	6
Substrate temp	MNS2-9	12	0–4095	60
Room temp	KSUM5	10	0–1095	6
Substrate hum	FC28YL38	12	0–4095	6
Room hum	KSUM5	10	0–1095	10
Red int	TSC34725	16	0–65,535	6
Green int	TSC34725	16	0–65,535	6
Blue int	TSC34725	16	0–65,535	6
Clear int	TSC34725	16	0–65,535	6
Photoperi	LDR	12	0–4095	6
En. cons	MNS29IN	12	0–4095	60
Camera	C920LOGI	1920 × 1080	0–255	30

**Table 3:** MSE for leaf length polynomial regression models.

Model	P1	P2	P3	P4	P5	P6	P7	P8	P9	P10	P11	P12
1	$2.13\times 10^{0}$	$4.91\times 10^{0}$	$1.12\times 10^{1}$	$1.64\times 10^{1}$	$1.79\times 10^{1}$	$1.35\times 10^{1}$	$1.26\times 10^{1}$	$1.16\times 10^{1}$	$5.36\times 10^{0}$	$4.07\times 10^{0}$	$2.71\times 10^{0}$	$1.45\times 10^{0}$
2	$9.57\times 10^{-1}$	$1.04\times 10^{0}$	$2.41\times 10^{0}$	$5.14\times 10^{0}$	$9.50\times 10^{0}$	$9.68\times 10^{0}$	$1.12\times 10^{1}$	$1.00\times 10^{1}$	$4.26\times 10^{0}$	$3.46\times 10^{0}$	$1.94\times 10^{0}$	$1.07\times 10^{0}$
3	$8.36\times 10^{-1}$	$7.65\times 10^{-1}$	$2.30\times 10^{0}$	$3.85\times 10^{0}$	$4.61\times 10^{0}$	$4.77\times 10^{0}$	$5.46\times 10^{0}$	$5.00\times 10^{0}$	$3.87\times 10^{0}$	$3.26\times 10^{0}$	$1.93\times 10^{0}$	$1.03\times 10^{0}$
4	$6.64\times 10^{-1}$	$4.29\times 10^{-1}$	$1.48\times 10^{0}$	$1.96\times 10^{0}$	$3.81\times 10^{0}$	$4.69\times 10^{0}$	$4.57\times 10^{0}$	$4.70\times 10^{0}$	$1.91\times 10^{0}$	$1.91\times 10^{0}$	$1.59\times 10^{0}$	$9.72\times 10^{-1}$
5	$5.47\times 10^{-1}$	$3.63\times 10^{-1}$	$1.41\times 10^{0}$	$1.48\times 10^{0}$	$1.69\times 10^{0}$	$2.09\times 10^{0}$	$3.66\times 10^{0}$	$3.06\times 10^{0}$	$6.35\times 10^{-1}$	$7.24\times 10^{-1}$	$8.54\times 10^{-1}$	$7.03\times 10^{-1}$
6	$4.07\times 10^{-1}$	$3.52\times 10^{-1}$	$1.19\times 10^{0}$	$1.45\times 10^{0}$	$1.58\times 10^{0}$	$1.67\times 10^{0}$	$2.00\times 10^{0}$	$1.50\times 10^{0}$	$3.93\times 10^{-1}$	$4.49\times 10^{-1}$	$3.39\times 10^{-1}$	$3.72\times 10^{-1}$
7	$3.82\times 10^{-1}$	$2.16\times 10^{-1}$	$1.19\times 10^{0}$	$1.44\times 10^{0}$	$1.57\times 10^{0}$	$1.49\times 10^{0}$	$1.78\times 10^{0}$	$1.50\times 10^{0}$	$3.77\times 10^{-1}$	$4.49\times 10^{-1}$	$1.97\times 10^{-1}$	$1.84\times 10^{-1}$
8	$4.17\times 10^{-1}$	$1.99\times 10^{-1}$	$8.69\times 10^{-1}$	$1.28\times 10^{0}$	$1.52\times 10^{0}$	$1.31\times 10^{0}$	$1.76\times 10^{0}$	$1.21\times 10^{0}$	$3.69\times 10^{-1}$	$4.02\times 10^{-1}$	$1.92\times 10^{-1}$	$1.46\times 10^{-1}$
9	$4.23\times 10^{-1}$	$1.86\times 10^{-1}$	$6.96\times 10^{-1}$	$9.08\times 10^{-1}$	$1.35\times 10^{0}$	$1.26\times 10^{0}$	$1.64\times 10^{0}$	$9.41\times 10^{-1}$	$3.55\times 10^{-1}$	$3.52\times 10^{-1}$	$1.90\times 10^{-1}$	$1.48\times 10^{-1}$
10	$5.19\times 10^{-1}$	$6.81\times 10^{-1}$	$7.82\times 10^{-1}$	$9.92\times 10^{0}$	$1.38\times 10^{0}$	$1.24\times 10^{0}$	$1.58\times 10^{0}$	$8.86\times 10^{-1}$	$3.56\times 10^{-1}$	$3.36\times 10^{-1}$	$1.90\times 10^{-1}$	$1.50\times 10^{-1}$
11	$5.03\times 10^{-1}$	$8.85\times 10^{-1}$	$1.30\times 10^{0}$	$1.02\times 10^{0}$	$1.25\times 10^{0}$	$1.24\times 10^{0}$	$1.47\times 10^{0}$	$8.41\times 10^{-1}$	$3.55\times 10^{-1}$	$3.08\times 10^{-1}$	$1.82\times 10^{-1}$	$1.44\times 10^{-1}$
12	$4.91\times 10^{-1}$	$1.03\times 10^{0}$	$2.09\times 10^{0}$	$1.49\times 10^{0}$	$1.18\times 10^{0}$	$1.24\times 10^{0}$	$1.39\times 10^{0}$	$8.41\times 10^{-1}$	$3.62\times 10^{-1}$	$2.85\times 10^{-1}$	$1.76\times 10^{-1}$	$1.30\times 10^{-1}$

**Table 4:** MSE for leaf width polynomial regression models.

Model	P1	P2	P3	P4	P5	P6	P7	P8	P9	P10	P11	P12
1	$5.13\times 10^{-1}$	$1.94\times 10^{0}$	$5.92\times 10^{0}$	$8.08\times 10^{0}$	$8.98\times 10^{0}$	$6.74\times 10^{0}$	$6.13\times 10^{0}$	$5.05\times 10^{0}$	$4.74\times 10^{0}$	$2.63\times 10^{0}$	$1.46\times 10^{0}$	$7.00\times 10^{-1}$
2	$2.01\times 10^{-1}$	$2.62\times 10^{-1}$	$1.36\times 10^{0}$	$2.59\times 10^{0}$	$4.99\times 10^{0}$	$5.92\times 10^{0}$	$5.78\times 10^{0}$	$5.05\times 10^{0}$	$3.61\times 10^{0}$	$1.91\times 10^{0}$	$1.04\times 10^{0}$	$5.20\times 10^{-1}$
3	$1.87\times 10^{-1}$	$2.52\times 10^{-1}$	$1.31\times 10^{0}$	$1.69\times 10^{0}$	$2.41\times 10^{0}$	$2.48\times 10^{0}$	$2.98\times 10^{0}$	$3.27\times 10^{0}$	$3.47\times 10^{0}$	$1.90\times 10^{0}$	$1.02\times 10^{0}$	$4.92\times 10^{-1}$
4	$1.47\times 10^{-1}$	$1.82\times 10^{-1}$	$7.22\times 10^{-1}$	$9.95\times 10^{-1}$	$2.05\times 10^{0}$	$2.01\times 10^{0}$	$2.11\times 10^{0}$	$1.41\times 10^{0}$	$2.06\times 10^{0}$	$1.40\times 10^{0}$	$9.03\times 10^{-1}$	$4.77\times 10^{-1}$
5	$1.11\times 10^{-1}$	$1.27\times 10^{-1}$	$6.60\times 10^{-1}$	$7.61\times 10^{-1}$	$8.72\times 10^{-1}$	$1.48\times 10^{0}$	$1.96\times 10^{0}$	$1.25\times 10^{0}$	$7.50\times 10^{-1}$	$6.15\times 10^{-1}$	$5.63\times 10^{-1}$	$3.68\times 10^{-1}$
6	$9.15\times 10^{-2}$	$1.11\times 10^{-1}$	$6.01\times 10^{-1}$	$7.26\times 10^{-1}$	$8.07\times 10^{-1}$	$6.73\times 10^{-1}$	$1.15\times 10^{0}$	$1.02\times 10^{0}$	$3.42\times 10^{-1}$	$2.11\times 10^{-1}$	$2.59\times 10^{-1}$	$2.12\times 10^{-1}$
7	$8.27\times 10^{-2}$	$9.05\times 10^{-2}$	$5.94\times 10^{-1}$	$6.84\times 10^{-1}$	$7.92\times 10^{-1}$	$5.91\times 10^{-1}$	$8.50\times 10^{-1}$	$5.63\times 10^{-1}$	$3.00\times 10^{-1}$	$1.46\times 10^{-1}$	$1.33\times 10^{-1}$	$1.06\times 10^{-1}$
8	$8.66\times 10^{-2}$	$6.62\times 10^{-2}$	$4.08\times 10^{-1}$	$6.58\times 10^{-1}$	$7.64\times 10^{-1}$	$5.85\times 10^{-1}$	$8.48\times 10^{-1}$	$4.09\times 10^{-1}$	$2.99\times 10^{-1}$	$1.47\times 10^{-1}$	$1.15\times 10^{-1}$	$7.62\times 10^{-2}$
9	$8.92\times 10^{-2}$	$6.50\times 10^{-2}$	$3.51\times 10^{-1}$	$4.71\times 10^{-1}$	$6.89\times 10^{-1}$	$5.62\times 10^{-1}$	$8.29\times 10^{-1}$	$3.85\times 10^{-1}$	$2.99\times 10^{-1}$	$1.43\times 10^{-1}$	$1.16\times 10^{-1}$	$7.41\times 10^{-2}$
10	$1.12\times 10^{-1}$	$1.86\times 10^{-1}$	$4.22\times 10^{-1}$	$4.97\times 10^{-1}$	$6.97\times 10^{-1}$	$5.46\times 10^{-1}$	$8.13\times 10^{-1}$	$3.84\times 10^{-1}$	$2.99\times 10^{-1}$	$1.41\times 10^{-1}$	$1.17\times 10^{-1}$	$7.57\times 10^{-2}$
11	$1.12\times 10^{-1}$	$2.97\times 10^{-1}$	$6.91\times 10^{-1}$	$4.26\times 10^{-1}$	$6.42\times 10^{-1}$	$5.22\times 10^{-1}$	$7.55\times 10^{-1}$	$3.84\times 10^{-1}$	$2.99\times 10^{-1}$	$1.37\times 10^{-1}$	$1.14\times 10^{-1}$	$7.51\times 10^{-2}$
12	$1.10\times 10^{-1}$	$3.96\times 10^{-1}$	$1.05\times 10^{0}$	$5.80\times 10^{-1}$	$6.12\times 10^{-1}$	$5.00\times 10^{-1}$	$6.95\times 10^{-1}$	$3.83\times 10^{-1}$	$2.98\times 10^{-1}$	$1.35\times 10^{-1}$	$1.09\times 10^{-1}$	$7.02\times 10^{-2}$

**Table 5:** MSE for leaf stem polynomial regression models in scientific notation.

Model	P1	P2	P3	P4	P5	P6	P7	P8	P9	P10	P11	P12
1	$2.56\times 10^{-1}$	$2.33\times 10^{0}$	$5.45\times 10^{0}$	$5.64\times 10^{0}$	$3.43\times 10^{0}$	$3.94\times 10^{0}$	$2.97\times 10^{0}$	$1.72\times 10^{0}$	$1.40\times 10^{0}$	$1.26\times 10^{0}$	$1.86\times 10^{-3}$	$1.86\times 10^{-3}$
2	$1.07\times 10^{-1}$	$2.18\times 10^{-1}$	$1.28\times 10^{0}$	$2.18\times 10^{0}$	$2.33\times 10^{0}$	$3.38\times 10^{0}$	$2.84\times 10^{0}$	$1.71\times 10^{0}$	$1.03\times 10^{0}$	$9.08\times 10^{-1}$	$1.50\times 10^{-3}$	$1.50\times 10^{-3}$
3	$8.38\times 10^{-2}$	$2.18\times 10^{-1}$	$1.06\times 10^{0}$	$1.63\times 10^{0}$	$9.68\times 10^{-1}$	$1.88\times 10^{0}$	$1.69\times 10^{0}$	$1.29\times 10^{0}$	$1.02\times 10^{0}$	$8.96\times 10^{-1}$	$1.41\times 10^{-3}$	$1.41\times 10^{-3}$
4	$6.91\times 10^{-2}$	$2.03\times 10^{-1}$	$6.62\times 10^{-1}$	$7.63\times 10^{-1}$	$9.56\times 10^{-1}$	$1.83\times 10^{0}$	$1.30\times 10^{0}$	$6.39\times 10^{-1}$	$6.80\times 10^{-1}$	$7.84\times 10^{-1}$	$1.41\times 10^{-3}$	$1.41\times 10^{-3}$
5	$5.43\times 10^{-2}$	$1.69\times 10^{-1}$	$6.25\times 10^{-1}$	$4.47\times 10^{-1}$	$4.17\times 10^{-1}$	$1.19\times 10^{0}$	$1.21\times 10^{0}$	$5.07\times 10^{-1}$	$2.65\times 10^{-1}$	$4.75\times 10^{-1}$	$1.25\times 10^{-3}$	$1.25\times 10^{-3}$
6	$4.26\times 10^{-2}$	$8.81\times 10^{-2}$	$5.27\times 10^{-1}$	$4.32\times 10^{-1}$	$3.19\times 10^{-1}$	$6.82\times 10^{-1}$	$6.80\times 10^{-1}$	$4.63\times 10^{-1}$	$9.96\times 10^{-2}$	$2.08\times 10^{-1}$	$9.22\times 10^{-4}$	$9.22\times 10^{-4}$
7	$3.71\times 10^{-2}$	$8.61\times 10^{-2}$	$4.91\times 10^{-1}$	$4.20\times 10^{-1}$	$3.13\times 10^{-1}$	$6.80\times 10^{-1}$	$5.11\times 10^{-1}$	$2.49\times 10^{-1}$	$8.23\times 10^{-2}$	$1.10\times 10^{-1}$	$5.93\times 10^{-4}$	$5.93\times 10^{-4}$
8	$3.97\times 10^{-2}$	$7.89\times 10^{-2}$	$3.99\times 10^{-1}$	$3.95\times 10^{-1}$	$3.05\times 10^{-1}$	$5.03\times 10^{-1}$	$5.15\times 10^{-1}$	$1.44\times 10^{-1}$	$8.24\times 10^{-2}$	$1.02\times 10^{-1}$	$4.41\times 10^{-4}$	$4.41\times 10^{-4}$
9	$3.86\times 10^{-2}$	$8.00\times 10^{-2}$	$2.72\times 10^{-1}$	$3.38\times 10^{-1}$	$2.95\times 10^{-1}$	$3.68\times 10^{-1}$	$4.39\times 10^{-1}$	$1.30\times 10^{-1}$	$8.19\times 10^{-2}$	$1.02\times 10^{-1}$	$4.05\times 10^{-4}$	$4.05\times 10^{-4}$
10	$6.08\times 10^{-2}$	$2.18\times 10^{-1}$	$2.80\times 10^{-1}$	$3.68\times 10^{-1}$	$2.91\times 10^{-1}$	$3.54\times 10^{-1}$	$4.17\times 10^{-1}$	$1.33\times 10^{-1}$	$8.18\times 10^{-2}$	$1.02\times 10^{-1}$	$4.09\times 10^{-4}$	$4.09\times 10^{-4}$
11	$5.90\times 10^{-2}$	$3.46\times 10^{-1}$	$3.86\times 10^{-1}$	$3.61\times 10^{-1}$	$2.83\times 10^{-1}$	$3.51\times 10^{-1}$	$3.47\times 10^{-1}$	$1.34\times 10^{-1}$	$8.17\times 10^{-2}$	$9.70\times 10^{-2}$	$4.16\times 10^{-4}$	$4.16\times 10^{-4}$
12	$5.70\times 10^{-2}$	$4.78\times 10^{-1}$	$6.84\times 10^{-1}$	$4.20\times 10^{-1}$	$2.72\times 10^{-1}$	$3.60\times 10^{-1}$	$3.14\times 10^{-1}$	$1.29\times 10^{-1}$	$8.15\times 10^{-2}$	$9.04\times 10^{-2}$	$4.08\times 10^{-4}$	$4.08\times 10^{-4}$

**Table 6:** MSE for Stem Diameter Polynomial Regression Models in scientific notation.

Model	P1	P2	P3	P4	P5	P6	P7	P8	P9	P10	P11	P12
1	$4.16\times 10^{-3}$	$5.78\times 10^{-3}$	$1.36\times 10^{-2}$	$2.74\times 10^{-2}$	$2.63\times 10^{-2}$	$2.34\times 10^{-2}$	$2.00\times 10^{-2}$	$1.88\times 10^{-2}$	$1.04\times 10^{-2}$	$7.82\times 10^{-3}$	$4.22\times 10^{-3}$	$1.86\times 10^{-3}$
2	$2.09\times 10^{-3}$	$1.40\times 10^{-3}$	$2.53\times 10^{-3}$	$9.31\times 10^{-3}$	$1.28\times 10^{-2}$	$1.89\times 10^{-2}$	$1.81\times 10^{-2}$	$1.88\times 10^{-2}$	$7.74\times 10^{-3}$	$5.61\times 10^{-3}$	$3.16\times 10^{-3}$	$1.50\times 10^{-3}$
3	$1.31\times 10^{-3}$	$9.92\times 10^{-4}$	$2.52\times 10^{-3}$	$6.71\times 10^{-3}$	$9.47\times 10^{-3}$	$9.91\times 10^{-3}$	$1.00\times 10^{-2}$	$1.32\times 10^{-2}$	$7.61\times 10^{-3}$	$5.57\times 10^{-3}$	$3.04\times 10^{-3}$	$1.41\times 10^{-3}$
4	$1.11\times 10^{-3}$	$9.69\times 10^{-4}$	$1.68\times 10^{-3}$	$3.72\times 10^{-3}$	$5.57\times 10^{-3}$	$9.87\times 10^{-3}$	$8.92\times 10^{-3}$	$7.06\times 10^{-3}$	$4.97\times 10^{-3}$	$4.66\times 10^{-3}$	$2.88\times 10^{-3}$	$1.41\times 10^{-3}$
5	$1.00\times 10^{-3}$	$5.01\times 10^{-4}$	$1.66\times 10^{-3}$	$2.71\times 10^{-3}$	$2.91\times 10^{-3}$	$5.45\times 10^{-3}$	$7.28\times 10^{-3}$	$6.59\times 10^{-3}$	$2.10\times 10^{-3}$	$2.56\times 10^{-3}$	$2.09\times 10^{-3}$	$1.25\times 10^{-3}$
6	$6.05\times 10^{-4}$	$4.94\times 10^{-4}$	$1.23\times 10^{-3}$	$2.66\times 10^{-3}$	$2.78\times 10^{-3}$	$3.33\times 10^{-3}$	$4.47\times 10^{-3}$	$5.29\times 10^{-3}$	$1.02\times 10^{-3}$	$1.04\times 10^{-3}$	$1.13\times 10^{-3}$	$9.22\times 10^{-4}$
7	$5.67\times 10^{-4}$	$3.75\times 10^{-4}$	$1.22\times 10^{-3}$	$2.65\times 10^{-3}$	$2.26\times 10^{-3}$	$3.19\times 10^{-3}$	$4.18\times 10^{-3}$	$2.67\times 10^{-3}$	$8.66\times 10^{-4}$	$6.43\times 10^{-4}$	$5.85\times 10^{-4}$	$5.93\times 10^{-4}$
8	$6.14\times 10^{-4}$	$4.03\times 10^{-4}$	$7.40\times 10^{-4}$	$2.35\times 10^{-3}$	$2.06\times 10^{-3}$	$2.25\times 10^{-3}$	$4.04\times 10^{-3}$	$1.90\times 10^{-3}$	$8.55\times 10^{-4}$	$6.40\times 10^{-4}$	$4.78\times 10^{-4}$	$4.41\times 10^{-4}$
9	$6.00\times 10^{-4}$	$5.91\times 10^{-4}$	$6.92\times 10^{-4}$	$1.73\times 10^{-3}$	$1.99\times 10^{-3}$	$1.79\times 10^{-3}$	$3.62\times 10^{-3}$	$1.92\times 10^{-3}$	$8.52\times 10^{-4}$	$6.24\times 10^{-4}$	$4.83\times 10^{-4}$	$4.05\times 10^{-4}$
10	$1.04\times 10^{-3}$	$1.52\times 10^{-3}$	$1.02\times 10^{-3}$	$1.89\times 10^{-3}$	$2.03\times 10^{-3}$	$1.75\times 10^{-3}$	$3.48\times 10^{-3}$	$1.98\times 10^{-3}$	$8.53\times 10^{-4}$	$6.19\times 10^{-4}$	$4.90\times 10^{-4}$	$4.09\times 10^{-4}$
11	$1.00\times 10^{-3}$	$1.74\times 10^{-3}$	$1.97\times 10^{-3}$	$1.92\times 10^{-3}$	$2.04\times 10^{-3}$	$1.77\times 10^{-3}$	$3.20\times 10^{-3}$	$1.88\times 10^{-3}$	$8.53\times 10^{-4}$	$5.90\times 10^{-4}$	$4.68\times 10^{-4}$	$4.16\times 10^{-4}$
12	$9.99\times 10^{-4}$	$1.86\times 10^{-3}$	$3.11\times 10^{-3}$	$2.60\times 10^{-3}$	$2.07\times 10^{-3}$	$1.77\times 10^{-3}$	$3.08\times 10^{-3}$	$1.67\times 10^{-3}$	$8.53\times 10^{-4}$	$5.73\times 10^{-4}$	$4.19\times 10^{-4}$	$4.08\times 10^{-4}$

**Table 7:** Polynomial coefficients and mean square errors for Length Leaf Pair (LLP).

LLP	Polynomial Coefficients P(x)	MSE
1	(0.95) + (1.17)x − (1.8 × $10^{-1}$)$x^{2}$ + (1 × $10^{-2}$)$x^{3}$− (6.4 × $10^{-4}$)$x^{4}$ + (1.9 × $10^{-5}$)$x^{5}$− (3.7 × $10^{-7}$)$x^{6}$ + (4.1 × $10^{-9}$)$x^{7}$− (1.9 × $10^{-11}$)$x^{8}$	0.3785
2	(0.17) + (0.03)x + (0.09)$x^{2}$− (1 × $10^{-2}$)$x^{3}$ + (1 × $10^{-3}$)$x^{4}$− (5.7 × $10^{-5}$)$x^{5}$ + (1.98 × $10^{-6}$)$x^{6}$− (4 × $10^{-8}$)$x^{7}$ + (4.2 × $10^{-10}$)$x^{8}$− (1.8 × $10^{-12}$)$x^{9}$	0.158
3	(0.23) − (1.7 × $10^{-2}$)x − (5.3 × $10^{-2}$)$x^{2}$ + (8.1 × $10^{-3}$)$x^{3}$ + (1.3 × $10^{-4}$)$x^{4}$− (5.3 × $10^{-5}$)$x^{5}$ + (2.8 × $10^{-6}$)$x^{6}$− (6.8 × $10^{-8}$)$x^{7}$ + (7.8 × $10^{-10}$)$x^{8}$− (3.5 × $10^{-12}$)$x^{9}$	0.5523
4	(−0.38) + (5.7 × $10^{-2}$)x + (0.17)$x^{2}$− (5.6 × $10^{-2}$)$x^{3}$ + (6.9 × $10^{-3}$)$x^{4}$− (4.1 × $10^{-4}$)$x^{5}$ + (1.3 × $10^{-5}$)$x^{6}$− (2.4 × $10^{-7}$)$x^{7}$ + (2.4 × $10^{-9}$)$x^{8}$− (9.4 × $10^{-12}$)$x^{9}$	0.832
5	(−0.63) + (6.8 × $10^{-2}$)x + (0.21)$x^{2}$− (5.9 × $10^{-2}$)$x^{3}$ + (6.4 × $10^{-3}$)$x^{4}$− (3.6 × $10^{-4}$)$x^{5}$ + (1.1 × $10^{-5}$)$x^{6}$− (2 × $10^{-7}$)$x^{7}$ + (2 × $10^{-9}$)$x^{8}$− (7.8 × $10^{-12}$)$x^{9}$	1.2153
6	(−0.29) + (2.7 × $10^{-2}$)x + (8.2 × $10^{-2}$)$x^{2}$− (2.3 × $10^{-2}$)$x^{3}$ + (2.6 × $10^{-3}$)$x^{4}$− (1.6 × $10^{-4}$)$x^{5}$ + (5.5 × $10^{-6}$)$x^{6}$− (1.1 × $10^{-7}$)$x^{7}$ + (1.1 × $10^{-9}$)$x^{8}$− (4.7 × $10^{-12}$)$x^{9}$	1.0742
7	(2.81 × $10^{-2}$)x + (8.46 × $10^{-2}$)$x^{2}$− (2.41 × $10^{-2}$)$x^{3}$ + (2.69 × $10^{-3}$)$x^{4}$− (1.54 × $10^{-4}$)$x^{5}$ + (4.91 × $10^{-6}$)$x^{6}$− (8.75 × $10^{-8}$)$x^{7}$ + (8.09 × $10^{-10}$)$x^{8}$− (3.02 × $10^{-12}$)$x^{9}$	1.5545
8	(−1.03 × $10^{-2}$)x − (3.10 × $10^{-2}$)$x^{2}$ + (8.81 × $10^{-3}$)$x^{3}$− (1.02 × $10^{-3}$)$x^{4}$ + (6.29 × $10^{-5}$)$x^{5}$− (2.26 × $10^{-6}$)$x^{6}$ + (4.73 × $10^{-8}$)$x^{7}$− (5.29 × $10^{-10}$)$x^{8}$ + (2.42 × $10^{-12}$)$x^{9}$	0.6681
9	(1.47 × $10^{-2}$)x + (4.42 × $10^{-2}$)$x^{2}$− (1.31 × $10^{-2}$)$x^{3}$ + (1.48 × $10^{-3}$)$x^{4}$− (8.53 × $10^{-5}$)$x^{5}$ + (2.74 × $10^{-6}$)$x^{6}$− (4.97 × $10^{-8}$)$x^{7}$ + (4.79 × $10^{-10}$)$x^{8}$− (1.91 × $10^{-12}$)$x^{9}$	0.3499
10	(−2.73 × $10^{-3}$)x − (8.16 × $10^{-3}$)$x^{2}$ + (2.72 × $10^{-3}$)$x^{3}$− (3.44 × $10^{-4}$)$x^{4}$ + (2.18 × $10^{-5}$)$x^{5}$− (7.57 × $10^{-7}$)$x^{6}$ + (1.44 × $10^{-8}$)$x^{7}$− (1.38 × $10^{-10}$)$x^{8}$ + (5.15 × $10^{-13}$)$x^{9}$	0.2228
11	(−1.07 × $10^{-2}$)x − (3.22 × $10^{-2}$)$x^{2}$ + (9.11 × $10^{-3}$)$x^{3}$− (9.93 × $10^{-4}$)$x^{4}$ + (5.51 × $10^{-5}$)$x^{5}$− (1.69 × $10^{-6}$)$x^{6}$ + (2.89 × $10^{-8}$)$x^{7}$− (2.54 × $10^{-10}$)$x^{8}$ + (8.87 × $10^{-13}$)$x^{9}$	0.1878
12	(1.81 × $10^{-1}$)x − (7.58 × $10^{-2}$)$x^{2}$ + (1.22 × $10^{-2}$)$x^{3}$− (9.64 × $10^{-4}$)$x^{4}$ + (4.11 × $10^{-5}$)$x^{5}$− (9.61 × $10^{-7}$)$x^{6}$ + (1.16 × $10^{-8}$)$x^{7}$− (5.61 × $10^{-11}$)$x^{8}$	0.1452

**Table 8:** Polynomial coefficients and mean square errors for Width Leaf Pair (WLP).

WLP	Polynomial Coefficients P(x)	MSE
1	(3.65 × $10^{-1}$) + (6.09 × $10^{-1}$)x − (1.1 × $10^{-1}$)$x^{2}$ + (1.2 × $10^{-2}$)$x^{3}$− (7.2 × $10^{-4}$)$x^{4}$ + (2.5 × $10^{-5}$)$x^{5}$− (5.2 × $10^{-7}$)$x^{6}$ + (5.75 × $10^{-9}$)$x^{7}$− (2.56 × $10^{-11}$)$x^{8}$	7.5 × $10^{-2}$
2	( − 2.52 × $10^{-2}$) + (1.65 × $10^{-2}$)x + (5.0 × $10^{-2}$)$x^{2}$ − (7.0 × $10^{-3}$)$x^{3}$ + (6.2 × $10^{-4}$)$x^{4}$ − (3.6 × $10^{-5}$)$x^{5}$ + (1.2 × $10^{-6}$)$x^{6}$ − (2.60 × $10^{-8}$)$x^{7}$ + (2.77 × $10^{-10}$)$x^{8}$ − (1.20 × $10^{-12}$)$x^{9}$	5.8 × $10^{-2}$
3	( − 3.51 × $10^{-1}$) + (9.27 × $10^{-1}$)x − (3.7 × $10^{-1}$)$x^{2}$ + (5.6 × $10^{-2}$)$x^{3}$ − (3.9 × $10^{-3}$)$x^{4}$ + (1.4 × $10^{-4}$)$x^{5}$ − (2.9 × $10^{-6}$)$x^{6}$ + (3.10 × $10^{-8}$)$x^{7}$ − (1.33 × $10^{-10}$)$x^{8}$	3.85 × $10^{-1}$
4	( − 3.06 × $10^{-1}$) + (4.24 × $10^{-2}$)x + (1.2 × $10^{-1}$)$x^{2}$ − (4.1 × $10^{-2}$)$x^{3}$ + (4.9 × $10^{-3}$)$x^{4}$ − (2.8 × $10^{-4}$)$x^{5}$ + (9.3 × $10^{-6}$)$x^{6}$ − (1.71 × $10^{-7}$)$x^{7}$ + (1.65 × $10^{-9}$)$x^{8}$ − (6.54 × $10^{-12}$)$x^{9}$	4.3 × $10^{-1}$
5	( − 4.39 × $10^{-1}$) + (4.66 × $10^{-2}$)x + (1.4 × $10^{-1}$)$x^{2}$ − (3.9 × $10^{-2}$)$x^{3}$ + (4.2 × $10^{-3}$)$x^{4}$ − (2.3 × $10^{-4}$)$x^{5}$ + (7.4 × $10^{-6}$)$x^{6}$ − (1.35 × $10^{-7}$)$x^{7}$ + (1.30 × $10^{-9}$)$x^{8}$ − (5.19 × $10^{-12}$)$x^{9}$	6.2 × $10^{-1}$
6	( − 1.88 × $10^{-1}$) + (1.96 × $10^{-2}$)x + (5.8 × $10^{-2}$)$x^{2}$ − (1.7 × $10^{-2}$)$x^{3}$ + (2.0 × $10^{-3}$)$x^{4}$ − (1.3 × $10^{-4}$)$x^{5}$ + (4.5 × $10^{-6}$)$x^{6}$ − (9.16 × $10^{-8}$)$x^{7}$ + (9.55 × $10^{-10}$)$x^{8}$ − (4.03 × $10^{-12}$)$x^{9}$	5.6 × $10^{-1}$
7	( − 4.18 × $10^{-1}$) + (4.43 × $10^{-2}$)x + (1.3 × $10^{-1}$)$x^{2}$ − (3.8 × $10^{-2}$)$x^{3}$ + (4.4 × $10^{-3}$)$x^{4}$ − (2.6 × $10^{-4}$)$x^{5}$ + (8.5 × $10^{-6}$)$x^{6}$ − (1.59 × $10^{-7}$)$x^{7}$ + (1.54 × $10^{-9}$)$x^{8}$ − (6.14 × $10^{-12}$)$x^{9}$	8.2 × $10^{-1}$
8	(5.07 × $10^{-2}$) − (6.16 × $10^{-3}$)x − (1.8 × $10^{-2}$)$x^{2}$ + (5.9 × $10^{-3}$)$x^{3}$ − (7.6 × $10^{-4}$)$x^{4}$ + (5.1 × $10^{-5}$)$x^{5}$ − (1.9 × $10^{-6}$)$x^{6}$ + (4.28 × $10^{-8}$)$x^{7}$ − (4.86 × $10^{-10}$)$x^{8}$ + (2.23 × $10^{-12}$)$x^{9}$	3.8 × $10^{-1}$
9	( − 1.68 × $10^{-2}$) + (2.02 × $10^{-3}$)x + (6.0 × $10^{-3}$)$x^{2}$ − (1.7 × $10^{-3}$)$x^{3}$ + (1.8 × $10^{-4}$)$x^{4}$ − (9.8 × $10^{-6}$)$x^{5}$ + (2.9 × $10^{-7}$)$x^{6}$ − (5.19 × $10^{-9}$)$x^{7}$ + (5.19 × $10^{-11}$)$x^{8}$ − (2.37 × $10^{-13}$)$x^{9}$	2.9 × $10^{-1}$
10	(8.09 × $10^{-2}$) − (8.71 × $10^{-3}$)x − (2.6 × $10^{-2}$)$x^{2}$ + (7.73 × $10^{-3}$)$x^{3}$ − (8.8 × $10^{-4}$)$x^{4}$ + (5.1 × $10^{-5}$)$x^{5}$ − (1.6 × $10^{-6}$)$x^{6}$ + (2.99 × $10^{-8}$)$x^{7}$ − (2.81 × $10^{-10}$)$x^{8}$ + (1.06 × $10^{-12}$)$x^{9}$	1.4 × $10^{-1}$
11	( − 3.82 × $10^{-2}$) + (1.00 × $10^{-1}$)x − (4.3 × $10^{-2}$)$x^{2}$ + (7.19 × $10^{-3}$)$x^{3}$ − (5.8 × $10^{-4}$)$x^{4}$ + (2.5 × $10^{-5}$)$x^{5}$ − (6.2 × $10^{-7}$)$x^{6}$ + (7.75 × $10^{-9}$)$x^{7}$ − (3.86 × $10^{-11}$)$x^{8}$	1.1 × $10^{-1}$
12	(2.72 × $10^{-2}$) − (1.48 × $10^{-3}$)x − (4.5 × $10^{-3}$)$x^{2}$ + (6.1 × $10^{-4}$)$x^{3}$ + (1.1 × $10^{-5}$)$x^{4}$ − (5.5 × $10^{-6}$)$x^{5}$ + (3.4 × $10^{-7}$)$x^{6}$ − (9.64 × $10^{-9}$)$x^{7}$ + (1.28 × $10^{-10}$)$x^{8}$ − (6.52 × $10^{-13}$)$x^{9}$	7.4 × $10^{-2}$

**Table 9:** Polynomial coefficients and mean square errors for Length Leaf Stem (LLS).

LLS	Polynomial Coefficients P(x)	MSE
1	(3.6 × $10^{-1}$) + (4.9 × $10^{-1}$)x − (9.5 × $10^{-2}$)$x^{2}$ + (1.0 × $10^{-2}$)$x^{3}$− (6.2 × $10^{-4}$)$x^{4}$ + (2.3 × $10^{-5}$)$x^{5}$− (4.7 × $10^{-7}$)$x^{6}$ + (5.1 × $10^{-9}$)$x^{7}$− (2.3 × $10^{-11}$)$x^{8}$	0.031
2	( − 2.5 × $10^{-2}$) + (1.5 × $10^{-3}$)x + (5.5 × $10^{-3}$)$x^{2}$ + (6.6 × $10^{-3}$)$x^{3}$− (8.6 × $10^{-4}$)$x^{4}$ + (4.6 × $10^{-5}$)$x^{5}$− (1.3 × $10^{-6}$)$x^{6}$ + (1.9 × $10^{-8}$)$x^{7}$− (1.4 × $10^{-10}$)$x^{8}$ + (3.9 × $10^{-13}$)$x^{9}$	0.076
3	( − 3.5 × $10^{-1}$) + (8.8 × $10^{-1}$)x − (3.5 × $10^{-1}$)$x^{2}$ + (5.1 × $10^{-2}$)$x^{3}$− (3.4 × $10^{-3}$)$x^{4}$ + (1.3 × $10^{-4}$)$x^{5}$− (2.5 × $10^{-6}$)$x^{6}$ + (2.7 × $10^{-8}$)$x^{7}$− (1.1 × $10^{-10}$)$x^{8}$	0.38
4	( − 2.4 × $10^{-1}$) + (3.1 × $10^{-2}$)x + (9.4 × $10^{-2}$)$x^{2}$− (2.9 × $10^{-2}$)$x^{3}$ + (3.2 × $10^{-3}$)$x^{4}$− (1.8 × $10^{-4}$)$x^{5}$ + (5.8 × $10^{-6}$)$x^{6}$− (1.0 × $10^{-7}$)$x^{7}$ + (1.0 × $10^{-9}$)$x^{8}$− (4.0 × $10^{-12}$)$x^{9}$	0.32
5	( − 1.7 × $10^{-1}$) + (1.7 × $10^{-2}$)x + (5.0 × $10^{-2}$)$x^{2}$− (1.3 × $10^{-2}$)$x^{3}$ + (1.4 × $10^{-3}$)$x^{4}$− (7.6 × $10^{-5}$)$x^{5}$ + (2.4 × $10^{-6}$)$x^{6}$− (4.5 × $10^{-8}$)$x^{7}$ + (4.5 × $10^{-10}$)$x^{8}$− (1.9 × $10^{-12}$)$x^{9}$	0.29
6	( − 3.2 × $10^{-1}$) + (3.6 × $10^{-2}$)x + (1.1 × $10^{-1}$)$x^{2}$− (3.3 × $10^{-2}$)$x^{3}$ + (3.9 × $10^{-3}$)$x^{4}$− (2.3 × $10^{-4}$)$x^{5}$ + (7.7 × $10^{-6}$)$x^{6}$− (1.5 × $10^{-7}$)$x^{7}$ + (1.4 × $10^{-9}$)$x^{8}$− (5.7 × $10^{-12}$)$x^{9}$	0.34
7	( − 3.7 × $10^{-1}$) + (4.1 × $10^{-2}$)x + (1.2 × $10^{-1}$)$x^{2}$− (3.7 × $10^{-2}$)$x^{3}$ + (4.2 × $10^{-3}$)$x^{4}$− (2.5 × $10^{-4}$)$x^{5}$ + (8.1 × $10^{-6}$)$x^{6}$− (1.5 × $10^{-7}$)$x^{7}$ + (1.4 × $10^{-9}$)$x^{8}$− (5.7 × $10^{-12}$)$x^{9}$	0.39
8	( − 1.5 × $10^{-2}$) − (6.5 × $10^{-4}$)x − (1.8 × $10^{-3}$)$x^{2}$ + (1.8 × $10^{-3}$)$x^{3}$− (3.5 × $10^{-4}$)$x^{4}$ + (3.0 × $10^{-5}$)$x^{5}$− (1.3 × $10^{-6}$)$x^{6}$ + (3.0 × $10^{-8}$)$x^{7}$− (3.6 × $10^{-10}$)$x^{8}$ + (1.7 × $10^{-12}$)$x^{9}$	0.13
9	(9.3 × $10^{-3}$) − (1.4 × $10^{-3}$)x − (4.3 × $10^{-3}$)$x^{2}$ + (1.5 × $10^{-3}$)$x^{3}$− (2.0 × $10^{-4}$)$x^{4}$ + (1.3 × $10^{-5}$)$x^{6}$− (4.5 × $10^{-7}$)$x^{6}$ + (8.7 × $10^{-9}$)$x^{7}$− (8.5 × $10^{-11}$)$x^{8}$ + (3.3 × $10^{-13}$)$x^{9}$	0.082
10	(8.8 × $10^{-2}$) − (8.5 × $10^{-3}$)x − (2.6 × $10^{-2}$)$x^{2}$ + (7.1 × $10^{-3}$)$x^{3}$− (7.4 × $10^{-4}$)$x^{4}$ + (4.0 × $10^{-5}$)$x^{5}$− (1.2 × $10^{-6}$)$x^{6}$ + (1.9 × $10^{-8}$)$x^{7}$− (1.6 × $10^{-10}$)$x^{8}$ + (5.1 × $10^{-13}$)$x^{9}$	0.1
11	(1.3 × $10^{-2}$) − (3.6 × $10^{-4}$)x − (1.1 × $10^{-3}$)$x^{2}$− (1.7 × $10^{-4}$)$x^{3}$ + (7.6 × $10^{-5}$)$x^{4}$− (8.0 × $10^{-6}$)$x^{5}$ + (3.8 × $10^{-7}$)$x^{6}$− (9.5 × $10^{-9}$)$x^{7}$ + (1.2 × $10^{-10}$)$x^{8}$− (5.8 × $10^{-13}$)$x^{9}$	0.056
12	( − 2.6 × $10^{-2}$) + (3.6 × $10^{-3}$)x + (1.1 × $10^{-2}$)$x^{2}$− (3.5 × $10^{-3}$)$x^{3}$ + (4.4 × $10^{-4}$)$x^{5}$− (2.8 × $10^{-5}$)$x^{6}$ + (9.9 × $10^{-7}$)$x^{7}$− (2.0 × $10^{-8}$)$x^{8}$ + (2.1 × $10^{-10}$)$x^{9}$	0.032

**Table 10:** Polynomial coefficients and mean square errors for Stem Diameter (SD).

WLS	Polynomial Coefficients P(x)	MSE
1	(4.0 × $10^{-2}$) + (5.6 × $10^{-2}$)x − (8.4 × $10^{-3}$)$x^{2}$ + (7.2 × $10^{-4}$)$x^{3}$− (4.0 × $10^{-5}$)$x^{4}$ + (1.4 × $10^{-6}$)$x^{5}$− (3.0 × $10^{-8}$)$x^{6}$ + (3.4 × $10^{-10}$)$x^{7}$− (1.6 × $10^{-12}$)$x^{8}$	5.0 × $10^{-4}$
2	( − 2.5 × $10^{-2}$) + (4.3 × $10^{-2}$)x − (3.7 × $10^{-3}$)$x^{2}$ + (2.9 × $10^{-4}$)$x^{3}$ − (2.1 × $10^{-5}$)$x^{4}$ + (9.8 × $10^{-7}$)$x^{5}$ − (2.4 × $10^{-8}$)$x^{6}$ + (3.0 × $10^{-10}$)$x^{7}$ − (1.5 × $10^{-12}$)$x^{8}$	3.0 × $10^{-4}$
3	(9.8 × $10^{-3}$) − (1.0 × $10^{-3}$)x − (3.0 × $10^{-3}$)$x^{2}$ + (5.9 × $10^{-4}$)$x^{3}$ − (2.1 × $10^{-5}$)$x^{4}$ − (8.6 × $10^{-7}$)$x^{5}$ + (7.8 × $10^{-9}$)$x^{6}$ − (2.1 × $10^{-10}$)$x^{7}$ + (2.6 × $10^{-13}$)$x^{8}$	7.0 × $10^{-4}$
4	( − 1.6 × $10^{-2}$) + (2.3 × $10^{-3}$)x + (7.0 × $10^{-3}$)$x^{2}$ − (2.3 × $10^{-3}$)$x^{3}$ + (2.8 × $10^{-4}$)$x^{4}$ − (1.7 × $10^{-5}$)$x^{5}$ + (5.4 × $10^{-7}$)$x^{6}$ − (1.0 × $10^{-8}$)$x^{7}$ + (9.7 × $10^{-11}$)$x^{8}$	1.6 × $10^{-3}$
5	( − 1.3 × $10^{-2}$) + (1.4 × $10^{-3}$)x + (4.1 × $10^{-3}$)$x^{2}$ − (1.2 × $10^{-3}$)$x^{3}$ + (1.2 × $10^{-4}$)$x^{4}$ − (6.9 × $10^{-6}$)$x^{5}$ + (2.2 × $10^{-7}$)$x^{6}$ − (4.2 × $10^{-9}$)$x^{7}$ + (4.2 × $10^{-11}$)$x^{8}$	1.9 × $10^{-3}$
6	( − 1.7 × $10^{-2}$) + (1.9 × $10^{-3}$)x + (5.7 × $10^{-3}$)$x^{2}$ − (1.8 × $10^{-3}$)$x^{3}$ + (2.1 × $10^{-4}$)$x^{4}$ − (1.3 × $10^{-5}$)$x^{5}$ + (4.4 × $10^{-7}$)$x^{6}$ − (8.5 × $10^{-9}$)$x^{7}$ + (8.5 × $10^{-11}$)$x^{8}$	1.7 × $10^{-3}$
7	( − 2.3 × $10^{-2}$) + (2.5 × $10^{-3}$)x + (7.5 × $10^{-3}$)$x^{2}$ − (2.2 × $10^{-3}$)$x^{3}$ + (2.5 × $10^{-4}$)$x^{4}$ − (1.5 × $10^{-5}$)$x^{5}$ + (4.8 × $10^{-7}$)$x^{6}$ − (8.8 × $10^{-9}$)$x^{7}$ + (8.5 × $10^{-11}$)$x^{8}$	3.5 × $10^{-3}$
8	(2.3 × $10^{-3}$) − (4.6 × $10^{-4}$)x − (1.4 × $10^{-3}$)$x^{2}$ + (5.1 × $10^{-4}$)$x^{3}$ − (7.2 × $10^{-5}$)$x^{4}$ + (5.0 × $10^{-6}$)$x^{5}$ − (1.9 × $10^{-7}$)$x^{6}$ + (4.2 × $10^{-9}$)$x^{7}$ − (4.7 × $10^{-11}$)$x^{8}$	1.4 × $10^{-3}$
9	( − 1.7 × $10^{-3}$) + (1.8 × $10^{-4}$)x + (5.5 × $10^{-4}$)$x^{2}$ − (1.6 × $10^{-4}$)$x^{3}$ + (1.7 × $10^{-5}$)$x^{4}$ − (9.6 × $10^{-7}$)$x^{5}$ + (3.0 × $10^{-8}$)$x^{6}$ − (5.6 × $10^{-10}$)$x^{7}$ + (5.7 × $10^{-12}$)$x^{8}$	9.0 × $10^{-4}$
10	(7.6 × $10^{-3}$) − (8.0 × $10^{-4}$)x − (2.4 × $10^{-3}$)$x^{2}$ + (6.8 × $10^{-4}$)$x^{3}$ − (7.5 × $10^{-5}$)$x^{4}$ + (4.2 × $10^{-6}$)$x^{5}$ − (1.3 × $10^{-7}$)$x^{6}$ + (2.3 × $10^{-9}$)$x^{7}$ − (2.1 × $10^{-11}$)$x^{8}$	6.0 × $10^{-4}$
11	( − 3.6 × $10^{-3}$) + (9.3 × $10^{-3}$)x − (3.9 × $10^{-3}$)$x^{2}$ + (6.4 × $10^{-4}$)$x^{3}$ − (5.1 × $10^{-5}$)$x^{4}$ + (2.2 × $10^{-6}$)$x^{5}$ − (5.1 × $10^{-8}$)$x^{6}$ + (6.2 × $10^{-10}$)$x^{7}$ − (3.0 × $10^{-12}$)$x^{8}$	5.0 × $10^{-4}$
12	( − 1.6 × $10^{-3}$) + (2.7 × $10^{-4}$)x + (8.0 × $10^{-4}$)$x^{2}$ − (2.8 × $10^{-4}$)$x^{3}$ + (3.6 × $10^{-5}$)$x^{4}$ − (2.4 × $10^{-6}$)$x^{5}$ + (8.8 × $10^{-8}$)$x^{6}$ − (1.8 × $10^{-9}$)$x^{7}$ + (1.9 × $10^{-11}$)$x^{8}$	4.0 × $10^{-4}$

## Data Availability

The data presented in this study are available on request from the corresponding author.

## References

[1] Hanif R., Iqbal Z., Iqbal M., Hanif S., Rasheed M. (2006). Use of vegetables as nutritional food: Role in human health. J. Agric. Biol. Sci..

[2] Ndlovu J., Afolayan A. (2008). Nutritional analysis of the South African wild vegetable Corchorus olitorius L. Asian J. Plant Sci.

[3] Kumar D., Kumar S., Shekhar C. (2020). Nutritional components in green leafy vegetables: A review. J. Pharmacogn. Phytochem..

[4] Zhang J., Sha Z., Zhang Y., Bei Z., Cao L. (2015). The effects of different water and nitrogen levels on yield, water and nitrogen utilization efficiencies of spinach (*Spinacia oleracea* L.). Can. J. Plant Sci..

[5] Beus C.E., Dunlap R.E. (1994). Agricultural Paradigms and the Practice of Agriculture 1. Rural. Sociol..

[6] Kassam A., Kassam L. (2021). Paradigms of agriculture. Rethinking Food and Agriculture.

[7] Tarakanov I., Yakovleva O., Konovalova I., Paliutina G., Anisimov A. (2012). Light-Emitting Diodes: On the way to Combinatorial Lighting Technologies for Basic Research and Crop production. Acta Hortic..

[8] Van Delden S., SharathKumar M., Butturini M., Graamans L., Heuvelink E., Kacira M., Kaiser E., Klamer R., Klerkx L., Kootstra G. (2021). Current status and future challenges in implementing and upscaling vertical farming systems. Nat. Food.

[9] Jürkenbeck K., Heumann A., Spiller A. (2019). Sustainability matters: Consumer acceptance of different vertical farming systems. Sustainability.

[10] Abul-Soud M., Mancy A. (2015). Urban horticulture of molokhia and spinach environmentally via green roof system and vermicomposting outputs. Global J. Adv. Res.

[11] Saad M.H.M., Hamdan N.M., Sarker M.R. (2021). State of the art of urban smart vertical farming automation system: Advanced topologies, issues and recommendations. Electronics.

[12] Araújo S.O., Peres R.S., Barata J., Lidon F., Ramalho J.C. (2021). Characterising the agriculture 4.0 landscape—emerging trends, challenges and opportunities. Agronomy.

[13] Chuah Y., Lee J., Tan S., Ng C. Implementation of smart monitoring system in vertical farming. Proceedings of the IOP Conference Series: Earth and Environmental Science, IOP Publishing.

[14] Park S.H., Lee J.H., Woo J.H., Choi S.Y., Park S.D., Moon Y.S. (2014). Control of Spinach Downy Mildew by Forced Ventilation in Greenhouse Cultivation. Korean J. Org. Agric..

[15] Piovene C., Orsini F., Bosi S., Sanoubar R., Bregola V., Dinelli G., Gianquinto G. (2015). Optimal red: Blue ratio in led lighting for nutraceutical indoor horticulture. Sci. Hortic..

[16] Ferrández-Pastor F.J., García-Chamizo J.M., Nieto-Hidalgo M., Mora-Pascual J., Mora-Martínez J. (2016). Developing ubiquitous sensor network platform using internet of things: Application in precision agriculture. Sensors.

[17] López-Riquelme J., Pavón-Pulido N., Navarro-Hellín H., Soto-Valles F., Torres-Sánchez R. (2017). A software architecture based on FIWARE cloud for Precision Agriculture. Agric. Water Manag..

[18] Wolfert S., Ge L., Verdouw C., Bogaardt M.J. (2017). Big data in smart farming–A review. Agric. Syst..

[19] Kamilaris A., Kartakoullis A., Prenafeta-Boldú F.X. (2017). A review on the practice of big data analysis in agriculture. Comput. Electron. Agric..

[20] Liakos K.G., Busato P., Moshou D., Pearson S., Bochtis D. (2018). Machine learning in agriculture: A review. Sensors.

[21] Tantalaki N., Souravlas S., Roumeliotis M. (2019). Data-driven decision making in precision agriculture: The rise of big data in agricultural systems. J. Agric. Food Inf..

[22] Torky M., Hassanein A.E. (2020). Integrating blockchain and the internet of things in precision agriculture: Analysis, opportunities, and challenges. Comput. Electron. Agric..

[23] Prasad R., Pandey A., Singh K., Singh V., Mishra R., Singh D. (2012). Retrieval of spinach crop parameters by microwave remote sensing with back propagation artificial neural networks: A comparison of different transfer functions. Adv. Space Res..

[24] Gent M.P. (2017). Factors affecting relative growth rate of lettuce and spinach in hydroponics in a greenhouse. HortScience.

[25] Torres I., Sánchez M.T., Vega-Castellote M., Luqui-Muñoz N., Pérez-Marín D. (2021). Routine NIRS analysis methodology to predict quality and safety indexes in spinach plants during their growing season in the field. Spectrochim. Acta Part Mol. Biomol. Spectrosc..

[26] Krisnawati M., Maftukah R., Rico R., Nadeak D., Nugroho B. Ameliorating Sandy Soil Properties: Application of Mathematical Model to Explore Spinach (*Amaranthus tricolor* L.) Plant Response. Proceedings of the IOP Conference Series: Earth and Environmental Science, IOP Publishing.

[27] Lu J., Tan L., Jiang H. (2021). Review on convolutional neural network (CNN) applied to plant leaf disease classification. Agriculture.

[28] Koyama K., Tanaka M., Cho B.H., Yoshikawa Y., Koseki S. (2021). Predicting sensory evaluation of spinach freshness using machine learning model and digital images. PLoS ONE.

[29] Islam M., Ria N.J., Ani J.F., Masum A.K.M., Abujar S., Hossain S.A. (2022). Deep Learning based classification system for recognizing local spinach. Proceedings of the Advances in Deep Learning, Artificial Intelligence and Robotics: Proceedings of the 2nd International Conference on Deep Learning, Artificial Intelligence and Robotics,(ICDLAIR) 2020.

[30] Sennan S., Pandey D., Alotaibi Y., Alghamdi S. (2022). A Novel Convolutional Neural Networks Based Spinach Classification and Recognition System. Comput. Mater. Contin..

[31] Saurer W., Possingham J. (1970). Studies on the growth of spinach leaves (Spinacea oleracea). J. Exp. Bot..

[32] Smolders E., Buysse J., Merckx R. (1993). Growth analysis of soil-grown spinach plants at different N-regimes. Proceedings of the Optimization of Plant Nutrition: Refereed papers from the Eighth International Colloquium for the Optimization of Plant Nutrition.

[33] Iwabuchi K., Goto E., Takakura T. (1996). Germination and growth of spinach under hypobaric conditions. Environ. Control. Biol..

[34] Kaminishi A., Kita N. (2006). Seasonal change of nitrate and oxalate concentration in relation to the growth rate of spinach cultivars. HortScience.

[35] Aisha H.A., Hafez M.M., Asmaa R.M., Shafeek M. (2013). Effect of Bio and chemical fertilizers on growth, yield and chemical properties of spinach plant (*Spinacia oleracea* L.). Middle East J. Agric. Res..

[36] Grevsen K., Kaack K. (1997). Quality attributes and morphological characteristics of spinach (*Spinacia oleracea* L.) cultivars for industrial processing. J. Veg. Crop. Prod..

[37] Matsuda R., Ohashi-Kaneko K., Fujiwara K., Kurata K. (2008). Effects of blue light deficiency on acclimation of light energy partitioning in PSII and CO2 assimilation capacity to high irradiance in spinach leaves. Plant Cell Physiol..

[38] Lim S.L., Wu T.Y., Lim P.N., Shak K.P.Y. (2015). The use of vermicompost in organic farming: Overview, effects on soil and economics. J. Sci. Food Agric..

[39] Lu C. (2015). Urban agriculture and vertical farming. Encycl. Sustain. Technol..

[40] Muianga C.A., Muniz J.A., Nascimento M.D.S., Fernandes T.J., Savian T.V. (2016). Description of the growth curve of cashew fruits in nonlinear models. Rev. Bras. Frutic..

[41] Ribeiro T.D., Mattos R.W.P.d., Morais A.R.d., Muniz J.A. (2018). Description of the growth of pequi fruits by nonlinear models. Rev. Bras. Frutic..

[42] Alhnaity B., Pearson S., Leontidis G., Kollias S. Using deep learning to predict plant growth and yield in greenhouse environments. Proceedings of the International Symposium on Advanced Technologies and Management for Innovative Greenhouses: GreenSys2019 1296.

